# Non-Targeted Metabolomic Profiling of Coronary Heart Disease Patients With Taohong Siwu Decoction Treatment

**DOI:** 10.3389/fphar.2020.00651

**Published:** 2020-05-08

**Authors:** Tianqi Tao, Tao He, Huimin Mao, Xudong Wu, Xiuhua Liu

**Affiliations:** ^1^Department of Pathophysiology, Chinese PLA General Hospital, Beijing, China; ^2^Outpatient Department, Chinese PLA General Hospital, Beijing, China

**Keywords:** metabolomics, coronary heart disease, Taohong Siwu decoction, UPLC-MS/MS, randomized double-blind trial

## Abstract

Traditional Chinese medicine is one of the complementary and alternative therapies to improve the prognosis of coronary heart disease (CHD). Taohong Siwu Decoction (THSWD), a classical traditional Chinese medication that promotes blood circulation, is clinically beneficial in CHD. However, the underlying mechanism of THSWD is still unclear. To comprehensively understand the material foundation of the “blood”, it is significantly important to study the differential metabolites involved in the treatment of CHD with Chinese medicinal herb promoting blood circulation in TCM theory. Hence, this study investigated the metabolic profiles of the serum in CHD patients to determine the differential metabolites between the THSWD group and the placebo group. Eleven CHD patients were recruited and divided into two groups randomly and double-blindly. Serum samples were determined by performing non-targeted ultra-performance liquid chromatography with tandem mass spectrometry-based metabolomics. Pearson’s correlation analysis was used to assess the association between identified metabolites and clinical serum indexes of CHD. Based on the result, a total of 513 metabolites were found in the serum of CHD patients, of which 27, involved in 29 metabolic pathways, were significantly different between the two groups. Among the differential metabolites, THSWD upregulated succinylcarnitine in fatty acid metabolism and 5′-methylthioadenosine in cysteine and methionine metabolism compared with the placebo group. However, THSWD downregulated pelargonic acid, involved in FA metabolism; succinate, involved in the tricarboxylic acid cycle; gluconic acid, gluconolactone, and d-glucose, involved in pentose phosphate pathway; glycerophosphocholine, involved in glycerophospholipid metabolism; 8,9-dihydroxyeicosatrienoic acid (8,9-DiHETrE), l-lysine, *N*-acetyl-l-aspartic acid, *N*-alpha-acetyl-l-asparagine, hippurate, indoxyl sulfate, and 3-ureidopropionate involved in amino acid metabolism compared with the placebo group. Moreover, succinylcarnitine, pelargonic acid, succinate, d-glucose, gluconic acid, l-lysine, *N*-alpha-acetyl-l-asparagine, 5′-methylthioadenosine, indoxyl sulfate, 8,9-DiHETrE, and 3-ureidopropionate were associated with total cholesterol or low-density lipoprotein. Succinylcarnitine, pelargonic acid, gluconolactone, *N*-acetyl-l-aspartic acid, *N*-alpha-acetyl-l-asparagine, hippurate, and 5′-methylthioadenosine were associated with activated partial thromboplastin time. Our findings indicated that glycerophosphocholine, 8,9-DiHETrE, 5′-methylthioadenosine, hippurate, indoxyl sulfate, and 3-ureidopropionate might constitute the partial material foundation of the “blood” in CHD patients treated with THSWD.

## Introduction

Coronary heart disease (CHD) is characterized by high morbidity and mortality, which contributes to cardiovascular diseases being the leading cause of death globally (Neumann et al., 2019). Therefore, it is crucial to concentrate on more effective treatment of CHD. In recent years, traditional Chinese medicine (TCM) has been considered a safe and effective alternative therapy in the treatment of CHD. TCM combined with Western medicine can be widely applied in CHD treatment. According to TCM theory, the blood is the mother of Qi, which means the blood is the carrier of Qi and governs nutrition to Qi. CHD with blood stasis syndrome is a common type of CHD; hence, promoting blood circulation and removing blood stasis in TCM theory are critical in CHD treatment (Xiong et al., 2015). Taohong Siwu Decoction (THSWD), originated from the “Golden Mirror of Medicine (Yizong Jinjian)” in Qing Dynasty, is commonly prescribed to promote blood circulation and remove blood stasis. It is composed of the following Chinese medicinal herbs: Tao Ren, Dang Gui, Chuan Xiong, Hong Hua, Chi Shao, and Sheng Di. According to the previous studies, THSWD improves angina symptoms, reduces blood lipids, and promotes anticoagulation (Ma et al., 2018; Tao et al., 2019). Other reports confirmed that THSWD improved microcirculation, protected the endothelial cells, and decreased the level of inflammatory factors (Liu et al., 2013). Although THSWD has beneficial effects on CHD treatment, its underlying mechanism is still unclear.

CHD is a complex metabolic disorder caused by coronary artery atherosclerosis, which leads to myocardial ischemia, infarction, or even heart failure. Metabolic abnormality had a critical role in CHD progression. Fatty acid (FA) oxidation is limited and the utilization of glucose catabolism increases in myocardial ischemia, which may eventually lead to the reduction of energy generation (Peterzan et al., 2017). Meanwhile, several accessory pathways of glucose metabolism such as the pentose phosphate pathway (PPP) are activated (Doenst et al., 2013). According to studies utilizing the acute blood stasis rat model and CHD patients with blood stasis syndrome, it is found that amino acid and lipid metabolites may be the partial material foundation of the “blood” in TCM theory (Hao et al., 2018; Zhao et al., 2019). Therefore, it is significantly important to study the differential metabolites to comprehensively understand the mechanism of Chinese medicinal herb promoting blood circulation in TCM theory.

Metabolomics (metabonomics) is considered an advanced systematic biological technique involved in the early screening, diagnosis, and prognosis of diseases by qualitatively and quantitatively analyzing low-molecular-weight metabolites (Shah et al., 2012). The most commonly used techniques of metabolomics mainly include high-resolution nuclear magnetic resonance spectroscopy and mass spectrometry (MS). MS is most commonly performed in conjunction with chromatography and is divided into gas chromatography-MS and liquid chromatography-MS (LC-MS). Among them, LC-MS is characterized by higher resolution and higher speed, which is appropriate to detect more complex samples in metabolomic studies. In this study, we used a non-targeted ultra-performance liquid chromatography with tandem mass spectrometry (UPLC-MS/MS) to study the differential low-molecular-weight metabolites in the serum of CHD patients before and after THSWD and placebo granules treatment to investigate the partial material foundation of the “blood”.

## Materials and Methods

### Recruitment and Study Design

**Figure 1:**
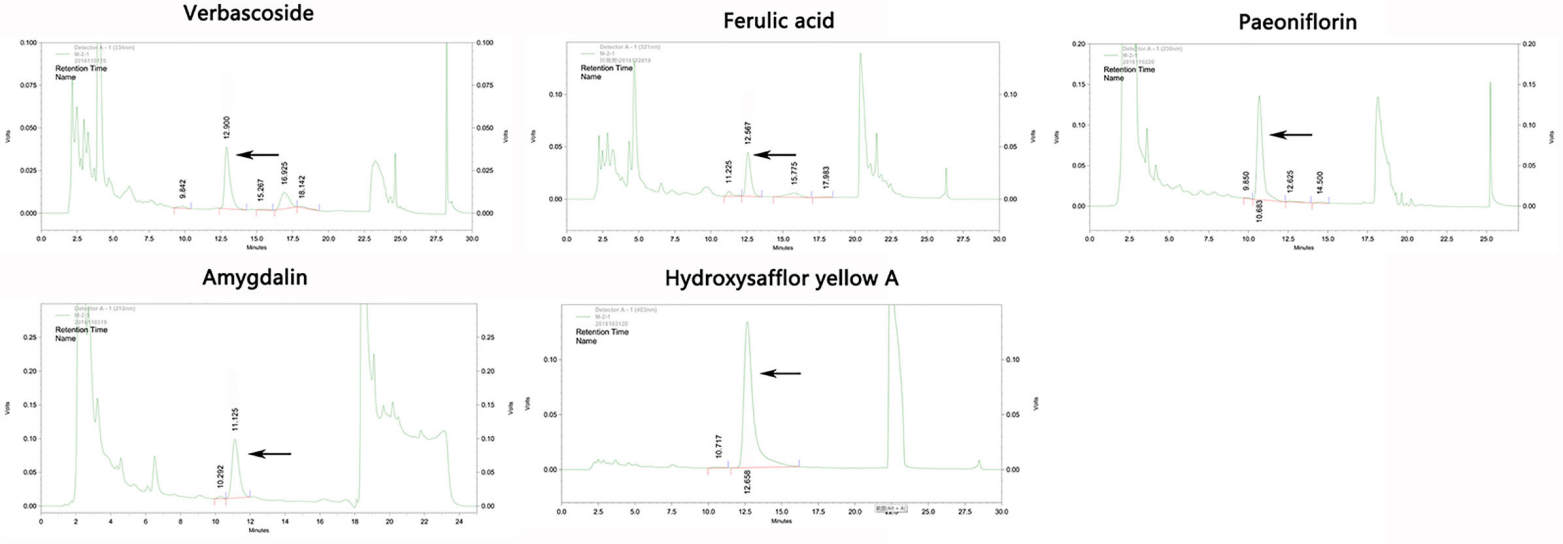
The high-performance liquid chromatography (HPLC) profile of the main chemical components in Taohong Siwu Decoction performed by the manufacturer.

**Figure 2:**
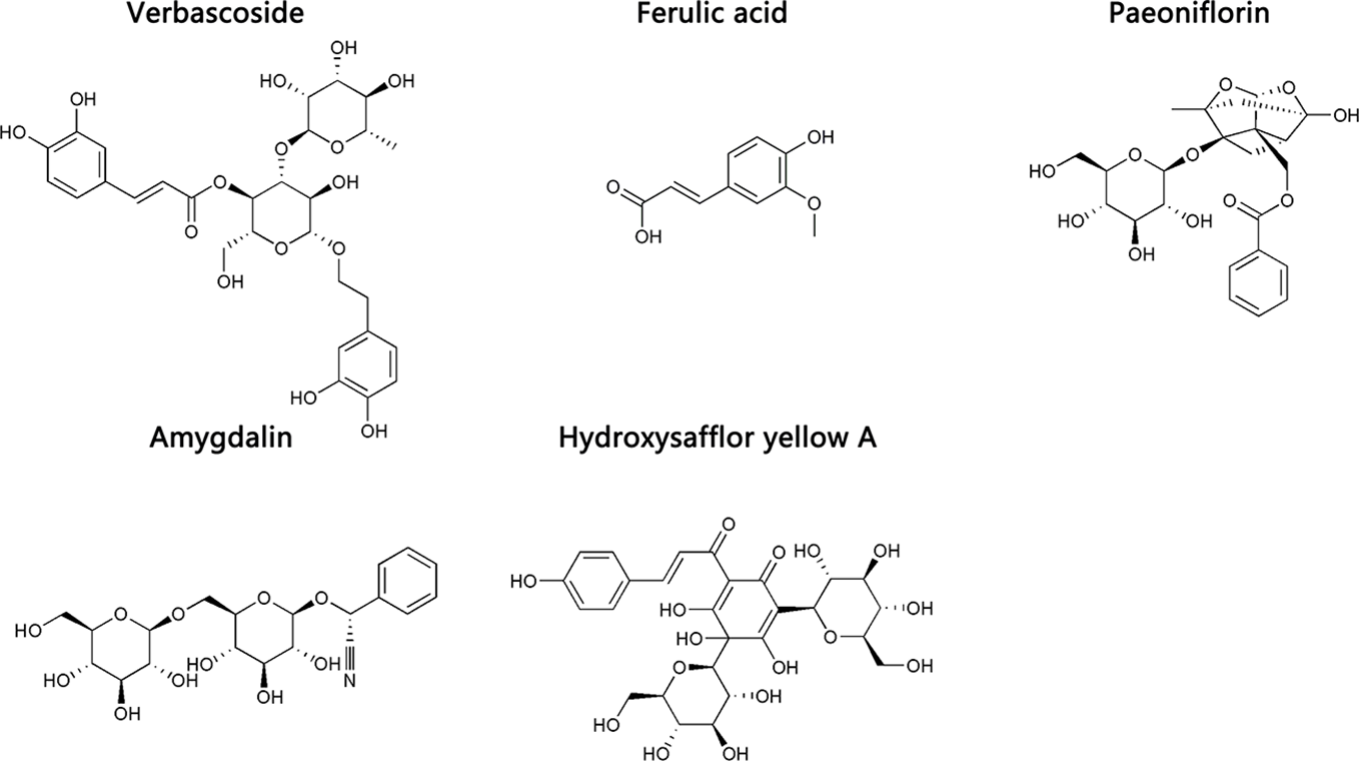
The chemical structures of the main chemical components of Taohong Siwu Decoction.

Eleven CHD patients were recruited in the Outpatient Department of the Chinese People’s Liberation Army (PLA) General Hospital from March 2017 to November 2017 in our study. CHD patients were treated according to the American College of Cardiology/American Heart Association (ACC/AHA) guidelines in 2014 (Fihn et al., 2014). Based on their routine Western medicine, five and six patients were treated with placebo granules for 12 weeks, and THSWD granules for 12 weeks, respectively. Demographic data were collected before the treatment, and clinical serum indexes were collected before and after the treatment. Placebo granules and THSWD granules were prepared from the China Resources Sanjiu Medical & Pharmaceutical Company (Shenzhen, China) according to the standard guidelines. Placebo granules were mainly composed of caramel (4 g) and maltodextrin (1000 g). The botanical compositions of THSWD granules are shown in **Table 1**, and the chemical compositions mainly consisted of verbascoside, ferulic acid, paeoniflorin, amygdalin, and hydroxysafflor yellow A, as indicated by the high-performance liquid chromatography (HPLC) profile of the extract performed by the manufacturer (**Figure 1**). The chemical structures of them were shown in **Figure 2**. The concentrations of the components in the three batches of THSWD samples detected by the manufacturer were listed in **Table 2**. The mean mass fractions of verbascoside, ferulic acid, paeoniflorin, amygdalin, and hydroxysafflor yellow A were 0.1003, 0.0280, 0.2792, 0.4790, and 0.3323 mg/g, respectively. Quality control (QC) of the pharmaceutical process was conducted in accordance with the Pharmaceutical Production Quality Management Standards (2015 edition). This study was approved by the Ethics Review Committee of the Chinese PLA General Hospital (No. S2015-048-01) and registered on the website of the Chinese Clinical Trial Registry (www.chictr.org.cn [Registration Number: ChiCTR-IOR-15006989]).

**Table 1:** Botanical compositions of Taohong Siwu Decoction.

Herb (Local Name)	Medicinal Parts	Amount in Application (g)
*Prunus persica* (L.) Batsch (Tao Ren)	Seed	12.5
*Angelica sinensis* (Oliv.) Diels (Dang Gui)	Root	9.375
*Conioselinum anthriscoides* “Chuanxiong” (Chuan Xiong)	Root	4.688
*Carthamus tinctorius* L. (Hong Hua)	Flower	9.375
*Paeonia lactiflora Pall*. (Chi Shao)	Root	6.25
*Rehmannia glutinosa* (Gaertn.) DC. (Sheng Di)	Root	9.375

All components in THSWD granules were validated using http://mpns.kew.org/mpns-portal/?_ga=1.111763972.1427522246.1459077346.

**Table 2:** Concentrations of the index components of Taohong Siwu Decoction (n = 3).

Lot Number	Mass fraction (mg·g^−1^)				
	Verbascoside	Ferulic Acid	Paeoniflorin	Amygdalin	Hydroxysafflor Yellow A
2016101801	0.0636	0.0181	0.2226	0.3731	0.2247
2016101802	0.1025	0.0281	0.1925	0.4401	0.3763
2016101803	0.1348	0.0376	0.4224	0.6239	0.3958
Mean	0.1003	0.0280	0.2792	0.4790	0.3323

The inclusion criteria for the diagnosis of CHD were as follows: patients younger than 75 years and patients who met the stable CHD diagnostic criteria issued by the ACC/AHA guidelines in 2014. Patients were treated with routine medicine according to the established guidelines, and the original treatment was maintained during the study. Complications were treated according to the relevant guidelines. Patients with the following characteristics were excluded in the study: patients with severe liver or kidney dysfunction, uncontrolled blood pressure, severe chronic heart failure, severe arrhythmia, cardiac infarction or cardiac pacemaker, diabetes mellitus, hemorrhagic diseases, malignant tumors, autoimmune diseases, and hematological or psychiatric diseases, pregnant or lactating women, and patients who may manifest a possible anaphylactic reaction to the research drugs’ components.

### Randomization, Control, and Double-Blinding

This was a double-blind, randomized, and placebo-controlled clinical trial. Participants were randomly grouped by researchers using the Statistical Analysis System statistical software according to a random number table. Drugs were coded and packaged by random numbers. The blind bottom could not be disassembled during the trial. All of the participants, staff, and researchers were blinded to the treatment group allocation.

### Sample Collection and Pre-treatment

Eleven participants fasted overnight, and 4 ml of peripheral venous blood was collected in the next morning and subsequently stored in a freezer at –80°C. Once the analysis was performed, the blood was thawed, and subsequently coagulated for 30 min at 4°C and centrifuged at 3000×*g* for 15 min. We added 400 μl of pre-chilled methanol to 100 μl serum supernatant; subsequently, the mixture was shaken for 15 s, incubated at −80°C for 1 h, and centrifuged at 13,400×*g* for 20 min at 4°C. The supernatant was dried before storage at −80°C. A pooled QC sample solution was prepared by combining equal volumes of the serum from each sample. Once one QC sample ended, the instrumental stability was controlled during the batch process (Li et al., 2018).

### Non-targeted Ultra-Performance Liquid Chromatography-Mass Spectrometry (MS)/MS Analysis and Metabolite Identification

Samples were detected by performing non-targeted UPLC-MS/MS using an Ultimate 3000 UHPLC (Dionex) system combined with a Thermo Q-Exactive (Orbitrap) mass spectrometer (Thermo Fisher Scientific, San Jose, CA, USA). Data identifications were performed using a Trace Finder. First, based on the endogenous MS database, metabolites were identified by accurate masses. Subsequently, the metabolites were identified at the MS/MS level using in-house MS/MS library, which was built using chemical standards. The matching confidence of experimental MS/MS spectra with MS/MS in the library was evaluated using library score. Normally, the metabolites with library score >30 were considered as MS/MS confirmed (Tang et al., 2016). The MS/MS spectra of representative metabolites were shown in the **Supplementary Figure**. A 0.25-min retention time deviation was applied, and the mass shifts of the primary and secondary identifications were 10 and 15 ppm, respectively (Ning et al., 2018).

### Data Processing

Multivariate statistical analysis was performed using the SIMCA 14.0 software. Unsupervised principal component analysis (PCA) was used to assess the quality, homogeneity, outlier identification, and dominating trends of the group separation inherent in the data set. A supervised orthogonal partial least squares discriminant analysis (OPLS-DA) was applied to determine the differences between the classes and to identify the differential metabolites. Variable Importance in the Projection (VIP) value was generated using the OPLS-DA model, and the quality of the multivariate statistical analysis model was evaluated using R^2^X and Q^2^. Student’s *t*-test was also used to determine the differences in metabolites between the two groups. Subsequently, the internal metabolite MS/MS database was used to identify metabolites by matching accurate quality and MS/MS spectra. MetaboAnalyst 4.0 (http://www.metaboanalyst.ca/) and Pearson’s correlation analysis were used to show the heat map of the differential metabolites and predict the metabolic pathways (Chong et al., 2018).

### Statistical Analysis

The Statistical Package for the Social Sciences version 17.0 (Chicago, IL, USA) was used for statistical analysis. Student’s *t*-test was performed for two-group comparisons on baseline characteristics and serum indexes before and after the treatment. Moreover, one-way analysis of variance was used to analyze the homogeneity of variance. Furthermore, values are presented as mean ± standard deviation. *P* < 0.05 was considered statistically significant.

## Results

### Clinical Characteristics

In our study, 11 CHD patients were divided into two groups randomly and double-blindly: five patients were treated with placebo granules, and six patients were treated with THSWD granules for 12 weeks. The baseline characteristics and serum indexes of the participants were shown in **Table 3**, which included gender, age, body mass index (BMI), blood pressure, heart rate, 5 kinds of blood routine indexes (including red blood cell, hemoglobin, white blood cell, neutrophil, and platelet), 10 kinds of biochemical indexes [including alanine aminotransferase, aspartate aminotransferase, serum creatinine, blood urea nitrogen, uric acid, triglyceride, total cholesterol (TC), low-density lipoprotein (LDL), high-density lipoprotein, and fasting glucose], and 4 kinds of coagulation parameters [including prothrombin time, activated partial thromboplastin time (APTT), fibrinogen, and thrombin time]. The average ages of the CHD patients in the placebo group and THSWD group were 55.60 ± 6.47 years and 59.83 ± 7.39 years, and the average BMIs in the placebo group and THSWD group were 25.55 ± 5.26 and 25.49 ± 2.79 kg/m^2^. The levels of TC and LDL were higher while that of APTT was lower in the THSWD group compared with the placebo group before the treatment (*P* < 0.05), while all values were within the normal ranges. However, there was no significant difference between the THSWD group and the placebo group in serum clinical indicators after the treatment (*P* > 0.05, **Table 4**), suggesting that THSWD granule treatment reversed the changes of the serum clinical indicators before treatment. It indicated that after THSWD granule treatment, levels of TC and LDL decreased while the level of the APTT increased in the THSWD group compared with the placebo group.

**Table 3:** Baseline characteristics of patients.

	Placebo (n = 5)	THSW (n = 6)	P
Gender (F/M)	1/4	3/3	0.545
Age, year	55.60 ± 6.47	59.83 ± 7.39	0.344
BMI, kg/m^2^	25.55 ± 5.26	25.49 ± 2.79	0.983
SBP, mm Hg	121.60 ± 16.70	132.33 ± 12.36	0.251
DBP, mm Hg	83.60 ± 9.94	77.67 ± 8.98	0.325
Heart rate, beats/min	70.40 ± 6.07	67.50 ± 7.53	0.506
RBC,×10^12^/L	4.71 ± 0.15	4.65 ± 0.54	0.805
HGB, g/L	140.40 ± 7.57	141.33 ± 18.10	0.912
WBC,×10^9^/L	6.06 ± 1.98	6.64 ± 1.02	0.544
NE, %	63.60 ± 9.72	58.92 ± 9.20	0.433
PLT,×10^9^/L	225.80 ± 43.34	219.67 ± 28.40	0.784
AST, U/L	22.38 ± 2.00	21.02 ± 5.72	0.604
ALT, U/L	25.60 ± 8.61	27.98 ± 19.50	0.807
SCr, μmol/L	75.98 ± 14.73	68.33 ± 18.87	0.480
BUN, mmol/L	5.05 ± 0.86	5.43 ± 1.01	0.524
UA, μmol/L	338.14 ± 87.40	365.48 ± 70.43	0.579
TG, mmol/L	1.97 ± 1.39	1.49 ± 0.60	0.465
TC, mmol/L	2.90 ± 1.43	4.73 ± 0.64*	0.020
HDL, mmol/L	1.30 ± 0.47	1.19 ± 0.29	0.631
LDL, mmol/L	1.96 ± 0.49	2.96 ± 0.31*	0.003
Glu, mmol/L	4.98 ± 0.56	5.42 ± 0.44	0.180
PT, s	13.12 ± 0.55	12.35 ± 0.83	0.112
APTT, s	37.42 ± 1.72	33.55 ± 2.50*	0.017
FIB, g/L	2.83 ± 0.34	2.80 ± 0.48	0.902
TT, s	16.42 ± 0.66	17.58 ± 2.92	0.409

Values are mean ± SD.

*P < 0.05 vs. the placebo group.

BMI, body mass index; SBP, systolic blood pressure; DBP, diastolic blood pressure; RBC, red blood cell; HGB, hemoglobin; WBC, white blood cell; NE, neutrophil; PLT, platelet; AST, aspartate aminotransferase; ALT, alanine aminotransferase; SCr, serum creatinine; BUN, blood urea nitrogen; UA, uric acid; TG, triglyceride; TC, total cholesterol; HDL, high-density lipoprotein; LDL, low-density lipoprotein; Glu, glucose; PT, prothrombin time; APTT, activated partial thromboplastin time; FIB, fibrinogen; TT, thrombin time.

**Table 4:** Clinical characteristics of patients after treatment.

	Placebo (n = 5)	THSW (n = 6)	P
RBC,×10^12^/L	5.00 ± 0.22	4.87 ± 0.49	0.573
HGB, g/L	149.00 ± 9.62	147.00 ± 15.09	0.804
WBC,×10^9^/L	5.48 ± 1.42	6.45 ± 1.46	0.296
NE, %	61.70 ± 8.84	56.38 ± 6.37	0.276
PLT,×10^9^/L	215.60 ± 48.24	217.83 ± 41.83	0.936
AST, U/L	22.70 ± 2.42	23.02 ± 8.65	0.939
ALT, U/L	24.82 ± 4.86	32.52 ± 24.80	0.515
SCr, μmol/L	77.70 ± 13.12	69.00 ± 16.43	0.365
BUN, mmol/L	4.75 ± 1.22	5.16 ± 1.15	0.583
UA, μmol/L	343.46 ± 107.60	316.40 ± 67.91	0.623
TG, mmol/L	1.40 ± 1.05	1.19 ± 0.44	0.650
TC, mmol/L	4.15 ± 0.39	4.82 ± 1.33	0.307
HDL, mmol/L	1.48 ± 0.53	1.21 ± 0.29	0.297
LDL, mmol/L	2.32 ± 0.50	3.20 ± 0.94	0.095
Glu, mmol/L	4.91 ± 0.53	5.27 ± 0.44	0.256
PT, s	12.90 ± 0.45	12.68 ± 0.40	0.419
APTT, s	37.24 ± 2.72	35.47 ± 3.20	0.354
FIB, g/L	2.98 ± 0.16	3.24 ± 0.26	0.092
TT, s	16.52 ± 0.93	16.52 ± 1.03	0.996

Values are mean ± SD. *P < 0.05 vs. the placebo group.

BMI, body mass index; SBP, systolic blood pressure; DBP, diastolic blood pressure; RBC, red blood cell; HGB, hemoglobin; WBC, white blood cell; NE, neutrophil; PLT, platelet; AST, aspartate aminotransferase; ALT, alanine aminotransferase; SCr, serum creatinine; BUN, blood urea nitrogen; UA, uric acid; TG, triglyceride; TC, total cholesterol; HDL, high-density lipoprotein; LDL, low-density lipoprotein; Glu, glucose; PT, prothrombin time; APTT, activated partial thromboplastin time; FIB, fibrinogen; TT, thrombin time.

### Analysis of Quality Control Samples

To investigate the partial material foundation of the “blood”, we detected serum metabolomics of CHD patients treated with THSWD by performing non-targeted LC-MS/MS analysis. We selected five serum samples in the placebo group and six serum samples in the THSWD group for metabolomic analysis at random. Our study used a customized database and non-targeted metabolomic methods to analyze the data. Furthermore, we identified hundreds of compounds from data collection to data analysis of clear compounds based on the established Orbitrap workflow. Therefore, a total of 513 metabolites were conformed in the samples before and after treatment, which were identified with known MS/MS information. The detailed information of 513 identified metabolites including compound name, electrospray ionization mode, mass, and retention time were shown in the **Supplementary Table**.

### Serum Metabolomics and Pathway Analysis

**Figure 3:**
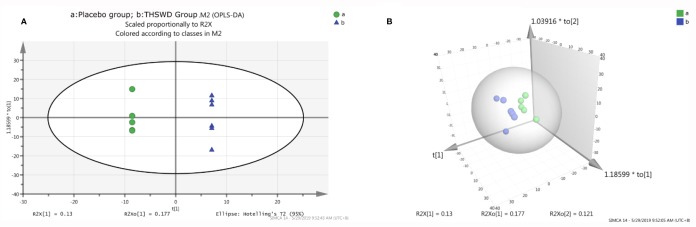
The orthogonal partial least structures discriminant analysis (OPLS-DA) score plots compared the placebo group **(A)** with the THSWD group **(B)**. The placebo group is shown in green color, and the THSWD group is shown in blue color. A: the two-dimensional graph of the OPLS-DA score plots. B: the three-dimensional graph of the OPLS-DA score plots.

A total of 513 metabolites were identified in our study, and these metabolites were imported into the SIMCA-P software for PCA. The result of PCA showed that there was no significant difference between the two groups (*P* > 0.05). We subsequently performed the analysis using the two- or three-dimensional orthogonal partial least-squares discriminant method obtained in an OPLS-DA score chart (**Figure 3**). It showed a significant difference between the THSWD group and the placebo group (R^2^X = 70.5%, Q^2^ = 57.9%). However, there was no statistically significant difference between the two groups after treatment, suggesting that there were some differences in the baseline level of differential metabolites, affecting the overall comparison results between the groups after treatment.

**Figure 4:**
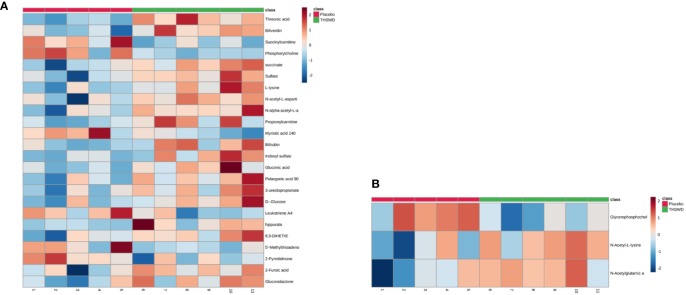
The 27 differential metabolites in the two groups are shown in the heat map using MetaboAnalyst 4.0 (**A**: 24 differential metabolites between the THSWD group and the placebo group before treatment; **B**: 3 differential metabolites between the THSWD group and the placebo group after treatment). The row represents the metabolites, and the column represents the individual samples. The deeper the color, the greater the difference in metabolites. Red bands indicate upregulated metabolites, and blue bands indicate downregulated metabolites in the two groups.

Considering the screening of identified metabolites under the conditions of VIP > 1 and *P* < 0.05, it was found that there were 24 kinds of differential metabolites in the THSWD group and the placebo group before the treatment (*P* < 0.05, VIP > 1), and the differences were not observed after 12 weeks of treatment. Additionally, there were three differential metabolites in the THSWD group and the placebo group after treatment (*P* < 0.05) with no statistically significant difference before treatment, suggesting that the treatment of THSWD also changed the level of other three differential metabolites, revealing that THSWD treatment changed a total of 27 differential metabolites (**Figure 4**, **Tables 5** and **6**).

**Table 5:** Differential metabolites between the THSWD group and the placebo group before treatment from UPLC-MS/MS analysis.

Differential metabolites	ESI mode	Mass (m/z)	Mass Shift (ppm)	MS/MS confirmed	RT (min)	VIP	P	FC (THSWD/placebo)
Threonic acid	neg	135.0299	-0.848793939	Yes	0.90	2.40042	0.00058	1.45
Biliverdin	pos	583.25511	-0.460942063	Yes	1.89	2.09742	0.007122	1.93
Succinylcarnitine	pos	262.12906	-2.25525746	Yes	9.46	2.07186	0.008251	0.66
Phosphorylcholine	pos	184.07332	-1.733257937	Yes	5.83	2.05796	0.008941	0.71
succinate	neg	117.01881	4.12066833	Yes	0.88	2.01993	0.011028	1.38
Sulfate	neg	96.9601	-0.4768798	Yes	0.87	1.99716	0.012423	1.79
L-lysine	neg	145.09773	3.31739348	Yes	1.02	1.93366	0.017102	1.77
N-acetyl-L-aspartic acid	neg	174.04027	3.13659881	Yes	0.88	1.89204	0.020726	1.60
N-alpha-acetyl-L-asparagine	neg	173.05626	2.43054742	No	18.92	1.87169	0.022759	1.48
Propionylcarnitine	pos	218.13868	-0.3028414	Yes	7.16	1.85991	0.023979	1.83
Myristic acid (14:0)	neg	227.20111	2.41737985	No	11.91	1.8426	0.025752	0.72
Bilirubin	neg	583.25621	0.49360338	No	10.55	1.82541	0.027776	1.58
Indoxyl sulfate	neg	212.00178	2.70346738	Yes	6.18	1.7993	0.030837	2.69
Gluconic acid	neg	195.0505	1.95042561	Yes	0.90	1.7919	0.031714	1.60
Pelargonic acid (9:0)	neg	157.1234	-0.3713829	No	8.78	1.77715	0.033688	1.43
3-ureidopropionate	neg	131.04569	3.22608662	No	0.93	1.75349	0.03689	1.41
D-(+)-Glucose	neg	179.05611	-0.2205941	No	1.00	1.7372	0.03926	1.43
Leukotriene A4	neg	317.21222	2.07733691	No	13.11	1.73305	0.039865	0.86
Hippurate	neg	178.05097	-0.0956546	Yes	4.85	1.71272	0.04291	4.27
8,9-DiHETrE	neg	337.23843	−7.429894	No	12.89	1.71191	0.043078	1.59
5′-Methylthioadenosine	pos	298.09736	8.90499169	No	11.32	1.69579	0.045691	0.60
2-Pyrrolidinone	pos	86.06004	0.77251831	Yes	1.40	1.69382	0.045915	0.68
2-Furoic acid	neg	111.00876	−0.369243	No	0.87	1.68541	0.047254	1.53
Gluconolactone	neg	177.03994	2.33424636	No	0.89	1.68089	0.047968	1.47

THSWD, Taohong Siwu Decoction; ESI, electrospray ionization; RT, retention time; VIP, variable importance in the projection; P, probability; FC, fold change; Pos, positive; Neg, negative.

The result of the heat map showed a total of 24 differential metabolites between the THSWD group and the placebo group before treatment (**Figure 4A**, **Table 5**). Before treatment, levels of the 18 differential metabolites in the THSWD group were higher compared with that of the placebo group. Biliverdin (fold change [FC] = 1.93, FC in the THSWD/placebo groups), and propionylcarnitine (FC = 1.83) were obtained by electrospray ionization-positive (ESI+) mode. Threonic acid (FC = 1.45), succinate (FC = 1.38), sulfate (FC = 1.79), l-lysine (FC = 1.77), *N*-acetyl-l-aspartic acid (FC = 1.60), *N*-alpha-acetyl-l-asparagine (FC = 1.48), bilirubin (FC = 1.58), indoxyl sulfate (FC = 2.69), gluconic acid (FC = 1.60), pelargonic acid (FC = 1.43), 3-ureidopropionate (3-UPA) (FC = 1.41), d-glucose (FC = 1.43), hippurate (FC = 4.27), 8,9-dihydroxyeicosatrienoic acid (8,9-DiHETrE) (FC = 1.59), 2-furoic acid (FC = 1.53), and gluconolactone (FC = 1.47) were obtained by electrospray ionization-negative (ESI–) mode. However, there was no significant difference among the above 18 metabolites after THSWD treatment for 12 weeks, suggesting that THSWD treatment downregulated these 18 metabolites. In the THSWD group before treatment, levels of the six differential metabolites were lower compared with that of the placebo group. Succinylcarnitine (FC = 0.66), phosphorylcholine (FC = 0.71), 5′-methylthioadenosine (5′-MTA) (FC = 0.60), and 2-pyrrolidinone (FC = 0.68) were obtained by ESI+ mode. Myristic acid (FC = 0.72) and leukotriene A4 (FC = 0.86) were obtained by ESI– mode. However, there was no significant difference among the above six metabolites after THSWD treatment, suggesting that THSWD treatment upregulated these six metabolites.

The analysis of another three metabolites showed no difference between the two groups before the treatment, but there was significant difference after the treatment (**Figure 4B**, **Table 6**). The results showed that in the THSWD group, glycerophosphocholine (FC = 0.67) was downregulated, and *N*-acetyl-l-lysine (FC = 1.21) and *N*-acetylglutamic acid (FC = 1.14) were upregulated.

**Table 6:** Differential metabolites between the THSWD group and the placebo group after treatment from UPLC-MS/MS analysis.

Differential metabolites	ESI mode	Mass (m/z)	Mass Shift (ppm)	MS/MS confirmed	RT (min)	P	FC (THSWD/placebo)
Glycerophosphocholine	pos	258.1101	−0.5460471	Yes	10.39	0.01205	0.67
*N*-acetyl-l-lysine	pos	189.12337	0.24395569	Yes	9.83	0.030883	1.21
*N*-acetylglutamic acid	neg	188.05645	−0.1722251	No	0.88	0.033159	1.14

THSWD, Taohong Siwu Decoction; ESI, electrospray ionization; RT, retention time; P, probability; FC, fold change; Pos, positive; Neg, negative.

**Figure 5:**
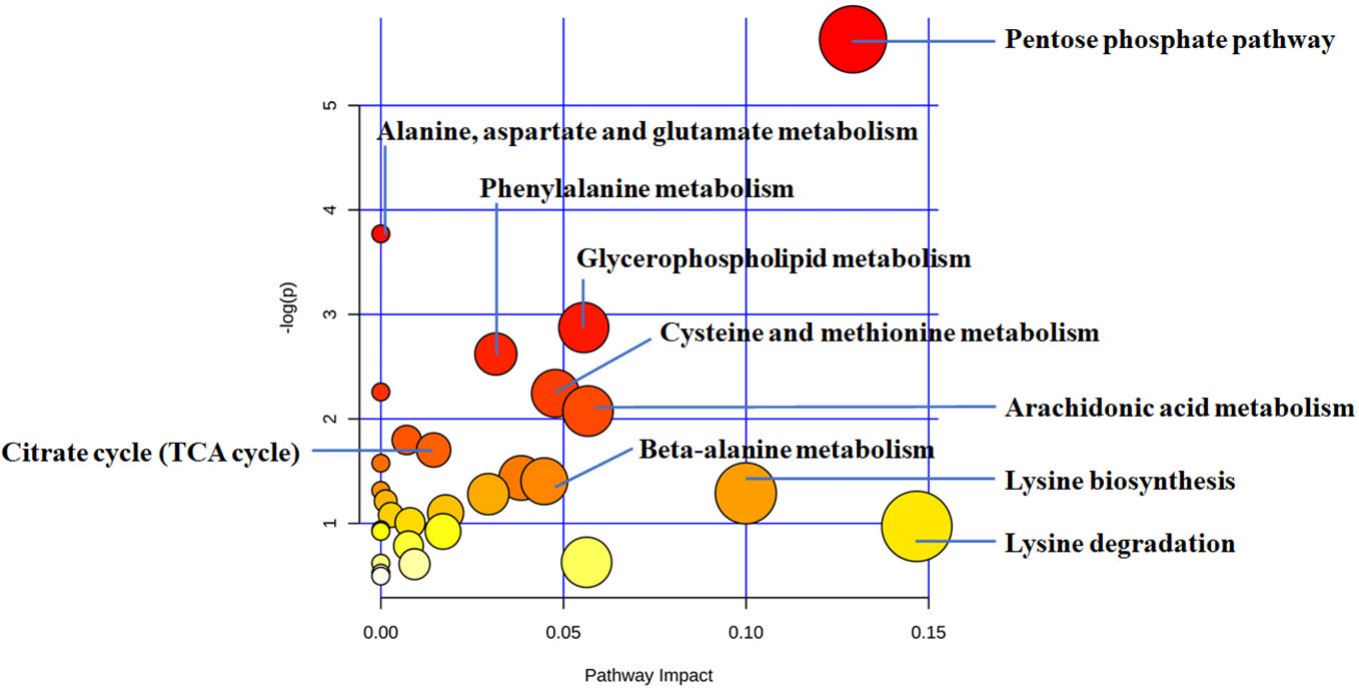
The disturbed metabolic pathways showed differential metabolites between the THSWD group and the placebo group by MetaboAnalyst 4.0 software. Node radius was based on pathway impact values. Node color was based on *P* value.

Considering software analysis and prediction, we found that the 27 differential metabolites associated with THSWD were involved in 29 metabolic pathways. The metabolic pathways closely associated with energy metabolism included FA metabolism, glycolysis or gluconeogenesis, citrate cycle (tricarboxylic acid [TCA] cycle), and PPP. Amino acid metabolism included phenylalanine metabolism; beta-alanine metabolism; alanine, aspartate, and glutamate metabolism; lysine biosynthesis; lysine degradation; cysteine and methionine metabolism; tyrosine metabolism; and arginine and proline metabolism. Additionally, it also included glycerophospholipid metabolism, arachidonic acid metabolism, biotin metabolism, sulfur metabolism, ether lipid metabolism, pantothenate and coenzyme A (CoA) biosynthesis, porphyrin and chlorophyll metabolism, propanoate metabolism, butanoate metabolism, galactose metabolism, ascorbate and aldarate metabolism, glyoxylate and dicarboxylate metabolism, starch and sucrose metabolism, pyrimidine metabolism, aminoacyl-transfer ribonucleic acid biosynthesis, amino sugar and nucleotide sugar metabolism, and purine metabolism (**Figure 5**, **Table 7**).

**Table 7:** Differential metabolic pathway between the THSWD group and the placebo group.

Pathway Name	Match Status	Match Metabolites
Pentose phosphate pathway	3/32	Gluconic acid; Gluconolactone; d-glucose
Alanine, aspartate, and glutamate metabolism	2/24	N-acetyl-l-aspartic acid; Succinic acid
Glycerophospholipid metabolism	2/39	Phosphorylcholine;Glycerophosphocholine
Phenylalanine metabolism	2/45	Hippuric acid; Succinic acid
Biotin metabolism	1/11	l-lysine
Cysteine and methionine metabolism	2/56	5′-Methylthioadenosine; Sulfate
Arachidonic acid metabolism	2/62	Leukotriene A4; 8,9-DiHETrE
Sulfur metabolism	1/18	Sulfate
Citrate cycle (TCA cycle)	1/20	Succinic acid
Ether lipid metabolism	1/23	Glycerophosphocholine
Pantothenate and CoA biosynthesis	1/27	Ureidopropionic acid
beta-Alanine metabolism	1/28	Ureidopropionic acid
Glycolysis or gluconeogenesis	1/31	d-Glucose
Lysine biosynthesis	1/32	l-Lysine
Porphyrin and chlorophyll metabolism	2/104	Biliverdin; Bilirubin
Propanoate metabolism	1/35	Succinic acid
Butanoate metabolism	1/40	Succinic acid
Galactose metabolism	1/41	d-Glucose
Ascorbate and aldarate metabolism	1/45	Threonic acid
Lysine degradation	1/47	l-lysine
Fatty acid biosynthesis	1/49	Myristic acid
Glyoxylate and dicarboxylate metabolism	1/50	Succinic acid
Starch and sucrose metabolism	1/50	d-Glucose
Pyrimidine metabolism	1/60	Ureidopropionic acid
Aminoacyl-tRNA biosynthesis	1/75	l-Lysine
Tyrosine metabolism	1/76	Succinic acid
Arginine and proline metabolism	1/77	N-Acetyl-l-alanine
Amino sugar and nucleotide sugar metabolism	1/88	d-Glucose
Purine metabolism	1/92	Sulfate

**Figure 6:**
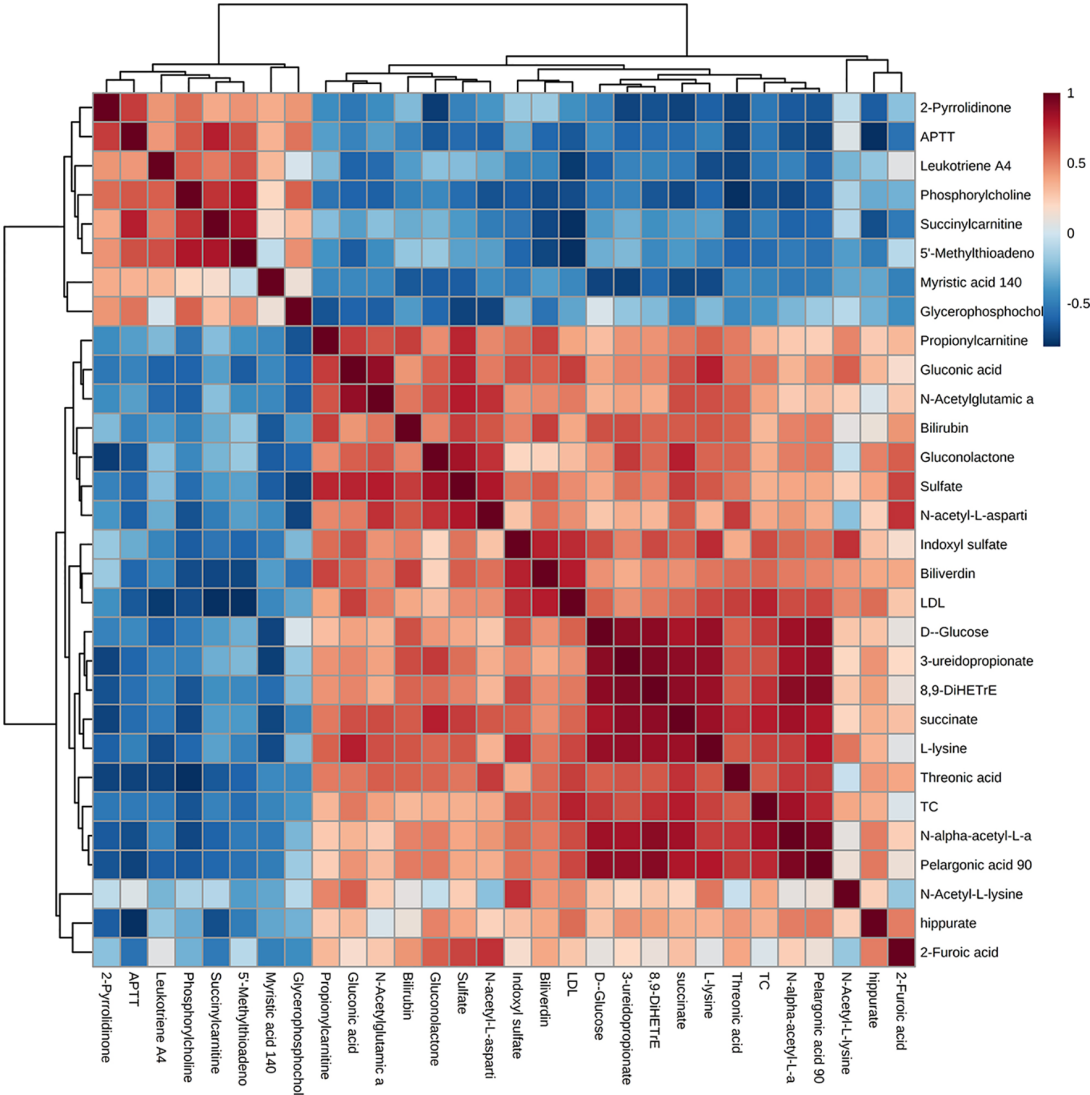
A Pearson’s correlation heat map of the serum metabolites and clinical parameters. Greater intensities of brown and blue indicate higher positive or negative correlations, respectively.

The correlation analysis between the 27 differential metabolites and differential serum clinical indexes (TC, LDL, and APTT) was shown by a Pearson’s correlation heat map (**Figure 6**). Among them, pelargonic acid (*P* = 0.008, R = 0.749), d-glucose (*P* = 0.017, R = 0.697), succinate (*P* = 0.004, R = 0.788), l-lysine (*P* = 0.022, R = 0.679), *N*-alpha-acetyl-l-asparagine (*P* = 0.001, R = 0.840), indoxyl sulfate (*P* = 0.029, R = 0.653), 8,9-DiHETrE (*P* = 0.010, R = 0.737), and 3-UPA (*P* = 0.035, R = 0.636) were positively associated with TC. Succinylcarnitine (*P* = 0.003, R = −0.801) and 5′-MTA (*P* = 0.003, R = −0.802) were negatively associated with LDL. Pelargonic acid (*P* = 0.027, R = 0.662), gluconic acid (*P* = 0.020, R = 0.685), l-lysine (*P* = 0.026, R = 0.664), *N*-alpha-acetyl-l-asparagine (*P* = 0.031, R = 0.648), and indoxyl sulfate (*P* = 0.009, R = 0.742) were positively associated with LDL. Succinylcarnitine (*P* = 0.005, R = 0.781) and 5′-MTA (*P* = 0.034, R = 0.639) were positively associated with APTT. Pelargonic acid (*P* = 0.010, R = −0.734), gluconolactone (*P* = 0.028, R=−0.658), *N*-acetyl-l-aspartic acid (*P* = 0.042, R = −0.620), *N*-alpha-acetyl-l-asparagine (*P* = 0.018, R = −0.694), and hippurate (*P* = 0.003, R=−0.800) were negatively associated with APTT.

## Discussion

Metabolic abnormality caused by myocardial ischemia plays a key role in the occurrence, development, and prognosis of CHD (Doenst et al., 2013). A combination of Western medicine and TCM is considered a safe and effective treatment for CHD. THSWD is a classical traditional Chinese medication that has a significant therapeutic effect on CHD by promoting blood circulation according to TCM theory. However, the underlying mechanism of THSWD on promoting blood circulation and removing blood stasis is unclear. Recent studies demonstrated that blood stasis was mainly associated with amino acid metabolism and lipid metabolism, suggesting that amino acid and lipid metabolites are associated with the “blood” in the TCM theory (Hao et al., 2018; Zhao et al., 2019). In this study, the non-targeted metabolomic analysis was used to detect the low-molecular-weight metabolites in CHD patients with THSWD and placebo granules treatment, and 513 kinds of metabolites were found. A total of 27 differential low-molecular-weight metabolites involved in 29 metabolic pathways were detected between the THSWD group and the placebo group. Among them, some metabolites play an important role on CHD. Succinylcarnitine in fatty acid metabolism and 5′-MTA in cysteine and methionine metabolism were increased in the THSWD group compared with the placebo group. However, pelargonic acid, involved in FA metabolism; succinate, involved in TCA cycle; gluconic acid, gluconolactone, and d-glucose, involved in PPP; glycerophosphocholine, involved in glycerophospholipid metabolism; 8,9-DiHETrE, involved in arachidonic acid metabolism; l-lysine, involved in lysine biosynthesis and degradation; *N*-acetyl-l-aspartic acid and *N*-alpha-acetyl-l-asparagine, involved in alanine, aspartate, and glutamate metabolism; hippurate, involved in phenylalanine metabolism; indoxyl sulfate, involved in tryptophan metabolism; and 3-UPA, involved in beta-alanine metabolism, were decreased in the THSWD group compared with the placebo group. Moreover, succinylcarnitine, pelargonic acid, succinate, d-glucose, gluconic acid, l-lysine, *N*-alpha-acetyl-l-asparagine, 5′-MTA, indoxyl sulfate, 8,9-DiHETrE, and 3-UPA were associated with TC or LDL. Succinylcarnitine, pelargonic acid, gluconolactone, *N*-acetyl-l-aspartic acid, *N*-alpha-acetyl-l-asparagine, hippurate, and 5′-MTA were associated with APTT. Our findings indicated that the low-molecular-weight metabolites might be associated with the anticoagulant and lipid-lowering effects of THSWD and constitute the partial material foundation of the “blood” in TCM theory.

### Lipid metabolism

#### Fatty Acid Metabolism

FA oxidation plays a critical role in myocardial energy generation. FAs are esterified to fatty acyl-CoA in the cytosol by carnitine palmitoyl transferase I (CPT I) located in the outer mitochondrial membrane. Long-chain acylcarnitine is subsequently transported into the mitochondrial matrix and converted back to long-chain acyl-CoA by CPT II. Acyl-CoA subsequently enters β-oxidation, generating acetyl-CoA (Peterzan et al., 2017). In this study, it was found for the first time that the serum level of succinylcarnitine in the THSWD group was lower compared with that of the placebo group before treatment, but there was no significant difference between the two groups after treatment with THSWD and placebo granules, suggesting that THSWD upregulated the succinylcarnitine level and promoted the β-oxidation of FAs. Further study showed that succinylcarnitine had a positive association with APTT and a negative association with LDL, illustrating that succinylcarnitine might be associated with the anticoagulant and lipid-lowering effects of THSWD.

Pelargonic acid, a type of medium-chain saturated FAs, provides free FAs for β-oxidation. A pilot study found that pelargonic acid was associated with the future onset of CHD in patients with type 2 diabetes by non-targeted metabolomic analysis (Omori et al., 2019). In our study, the serum level of pelargonic acid in the THSWD group was higher than that in the placebo group before treatment. However, after treatment, the changes in pelargonic acid were reversed, suggesting that THSWD may decrease the serum level of pelargonic acid by promoting the β-oxidation of FAs. Furthermore, it was found for the first time through correlation analysis that pelargonic acid was negatively associated with APTT, and positively associated with LDL and TC, suggesting that pelargonic acid might be related to the anticoagulation and lipid-lowering effects of THSWD.

#### Glycerophospholipid Metabolism

Phospholipids are involved in several pathophysiological process of CHD such as apoptosis, autophagy, proliferation, and inflammation. Phosphatidylcholine (PC) is one of the most important phospholipids in eukaryotic cells (Kosmas et al., 2018). LysoPC is the main component of ox-LDL. In the process of LDL oxidation, the level of lysoPC increases, and lysoPC is involved in the pathophysiological process of coronary atherosclerosis (Boyanovsky and Webb, 2009; Senn et al., 2012). Basak et al. found that lysoPC was better upregulated in the plasma of CHD patients than that of controls using LC-MS analysis (Basak et al., 2015). Glycerophosphocholine is produced by lysoPC. According to Hao et al.’s acute blood stasis rat model used to detect plasma metabolomics, the plasma level of glycerophosphocholine increased due to acute blood stasis (Hao et al., 2018). Our study showed that THSWD granule treatment downregulated glycerophosphocholine, suggesting that THSWD may alleviate coronary atherosclerosis and the symptoms of blood stasis by downregulating glycerophosphocholine.

#### Arachidonic Acid Metabolism

8,9-DiHETrE is a cytochrome P450 eicosanoid that is involved in the regulation of vascular tension, cardiac contractility, cell proliferation, and inflammation. The decrease of 8,9-DiHETrE indicates the limitation of the inflammation regulated by arachidonic acid. A recent cross-sectional study confirmed that 8,9-DiHETrE was significantly associated with cardiovascular events; with every 1 nmol/L increase in the concentrations of 8,9-DiHETrE, the odds of acute coronary syndrome (ACS) increased by 454 times, suggesting that 8,9-DiHETrE is the most significant predictor of ACS (Caligiuri et al., 2017; Solati and Ravandi, 2019). In this study, we found that the serum level of 8,9-DiHETrE in the THSWD group before treatment was higher than that in the placebo group, but there was no significant difference in 8,9-DiHETrE after the treatment between the THSWD group and the placebo group, indicating that THSWD may downregulate the serum level of 8,9-DiHETrE and arachidonic acid metabolism-mediated inflammation. Additionally, we initially found that 8,9-DiHETrE was positively associated with TC by correlation analysis, illustrating that THSWD might also reduce the serum level of TC by decreasing 8,9-DiHETrE level.

### Glucose Metabolism

The utilization of FAs and glucose is closely co-regulated in myocardial metabolism. The oxidation of FAs or glucose may directly inhibit the utilization of another, which is called “Randle cycle” (Randle, 1998). In myocardial ischemia, the oxidation of FAs is decreased, while glucose metabolism is increased in cardiomyocytes.

#### Tricarboxylic Acid Cycle

Pyruvate, generated through glycolysis, enters the mitochondrial matrix and undergoes oxidation to acetyl-CoA by the multienzyme complex pyruvate dehydrogenase. Subsequently, acetyl-CoA enters the TCA cycle and produced adenosine triphosphate (ATP). Succinate is the intermediate product of the tricarboxylic acid cycle. The formation of succinate is the only step to directly produce high-energy phosphate bond in the tricarboxylic acid cycle, which belongs to the substrate-level phosphorylation reaction. The succinate in the tricarboxylic acid cycle of the THSWD group was better up-regulated than that of the placebo group before treatment, but there was no statistically significant difference between the two groups after treatment, suggesting that the succinate was down-regulated after the treatment of THSWD. Combined with the changes in FA metabolism-related molecules, it suggests that glucose metabolism is inhibited and the intermediate product of the TAC cycle decreases due to the upregulation of FA metabolism. THSWD promoted energy generation with the increase of fatty acid metabolism and the decrease of glucose metabolism. Further correlation analysis showed that succinate was positively associated with TC, suggesting that THSWD could downregulated cholesterol level by reducing succinate level.

#### Pentose Phosphate Pathway

Glucose-6-phosphate is also possibly involved in PPP. PPP is an essential source of nicotinamide adenine dinucleotide phosphate (NADPH), which regulates oxidative stress and lipid synthesis and complement. In myocardial ischemia, the PPP is upregulated (Doenst et al., 2013). In this study, we found that levels of gluconic acid, gluconolactone, and d-glucose involved in PPP were higher than those in the placebo group before THSWD treatment, but there was no significant difference after treatment, suggesting that THSWD downregulates the levels of gluconic acid, gluconolactone, and d-glucose involved in PPP. Combined with the differential metabolites’ change in FA metabolism and the TCA cycle, it suggests that the upregulation of energy generation might inhibit PPP pathway. Furthermore, by performing correlation analysis, it was found for the first time that there was a negative association between gluconolactone and APTT; gluconic acid was positively associated with LDL; and there was a positive association between d-glucose and TC, suggesting that these three metabolites involved in PPP were also associated with the anticoagulant and lipid-lowering effects of THSWD.

### Amino Acid Metabolism

Previous studies have found that blood stasis is associated with amino acid metabolism, as shown by the decrease of tryptophan and the increase of tyrosine in the blood stasis group compared with the control group, suggesting that the “blood” in the theory of Qi and the blood in TCM are closely associated with amino acid metabolism (Hao et al., 2018; Zhao et al., 2019). Fan et al. revealed that plasma metabolomic profiles in patients with different types of CHD by performing liquid chromatography-quadrupole time-of-flight MS, and found that levels of the amino acid metabolites, such as tryptophan, arginine, and leucine, increased with CHD progression (Fan et al., 2016). However, based on a mini-swine model of progressive chronic heart failure, metabolomic analysis of ischemic tissue and plasma in the model group revealed a significant decrease of alanine and glycine involved in amino acid metabolism as compared with controls (Wang et al., 2013). For the first time, we found that THSWD downregulated l-lysine, *N*-acetyl-l-aspartic acid, and *N*-alpha-acetyl-l-asparagine, but upregulated *N*-acetyl-l-lysine, and *N*-acetylglutamic acid, suggesting that THSWD regulated amino acid metabolism. We also found that *N*-acetyl-l-aspartic acid and *N*-alpha-acetyl-l-asparagine were negatively associated with APTT. l-lysine and *N*-alpha-acetyl-l-asparagine was positively associated with TC and LDL. The results indicated that THSWD might play a critical role in lipid reduction and anticoagulation by regulating amino acid metabolism. The following small molecular metabolites may be closely associated with the mechanism of THSWD in the treatment of CHD.

5′-MTA, a nucleoside produced by S-adenosylmethionine during the synthesis of polyamines, is previously considered to inhibit tumor cell proliferation, invasion and apoptosis, and to regulate the inflammatory microenvironment of the tumor. Another study indicated that 5′-MTA pre-treatment significantly alleviated liver ischemia/reperfusion injury by inhibiting inflammatory response following liver transplantation *in vivo*, with the inhibition of NF-κB and MAPK signal pathway (Tang et al., 2014). We found that the level of 5′-MTA involved in cysteine and methionine metabolism was lower in the THSWD group than that in the placebo group before treatment; however, there was no significant difference in 5′-MTA level between the two groups after treatment, suggesting that THSWD upregulated the serum level of 5′-MTA. Further correlation analysis showed that 5′-MTA was positively associated with APTT and negatively associated with LDL, suggesting that 5′-MTA might be associated with the anticoagulation and lipid-lowering effect of THSWD.

Hippurate is a protein-bound uremic retention solute, which is significantly increased in the serum of patients with chronic renal failure (Vanholder et al., 2003). Previous studies found that hippurate levels in the serum were positively associated with diastolic blood pressure and echocardiographic indexes such as end-diastolic interventricular septal thickness, suggesting that hippurate can be considered as a new biomarker of the left ventricular overload (Yu et al., 2018). Additionally, other studies reported that hippurate causes endothelial dysfunction and oxidative stress by an increase in mitoROS *via* mitochondrial fission (Huang et al., 2018). We found that the level of hippurate in the THSWD group was higher than that in the placebo group before treatment, but there was no significant difference between the two groups after treatment, suggesting that THSWD decreases the serum level of hippurate. Furthermore, it was found for the first time through correlation analysis that hippurate was negatively associated with APTT, suggesting that hippurate might be associated with the anticoagulant effect of THSWD.

Indoxyl sulfate is also a protein-bound uremic toxin generated by tryptophan metabolism *via* the gut microbiota. A recent study found that indoxyl sulfate activated oxidative stress by regulating multiple NADPH oxidase-mediated redox signaling pathways and aggravated the progress of cardiovascular diseases such as chronic heart failure, arrhythmia, and CHD (Gao and Liu, 2017). Other studies prospectively assessed the all-cause mortality of 351 patients undergoing percutaneous revascularization for CHD or peripheral artery disease and found that the highest mortality rate was observed in the high indoxyl sulfate and low albumin group, suggesting that a higher indoxyl sulfate level add potentiating effects on lower albumin as a prognostic factor for cardiovascular disease (Watanabe et al., 2019). This study found that the level of indoxyl sulfate in the THSWD group was higher than that in the placebo group before treatment, but there was no significant difference between the two groups after treatment, suggesting that the level of indoxyl sulfate could be decreased after THSWD treatment. Further correlation analysis showed for the first time that indoxyl sulfate was positively associated with LDL and TC, suggesting that indoxyl sulfate might be associated with the lipid-lowering effect of THSWD.

3-UPA is a physiological metabolite produced by pyrimidine degradation that is involved in β-alanine metabolism. Other studies reported that 3-UPA acts as an endogenous neurotoxin by inhibiting mitochondrial energy metabolism. In cultured chick neurons, 3-UPA induced the increase of reactive oxygen species and intracellular calcium concentrations. 3-UPA also inhibited complex V activity in a single respiratory chain, but it did not affect the mitochondrial FA oxidation (Kölker et al., 2001). This study found that before the treatment the serum level of 3-UPA in the THSWD group was higher than that in the placebo group, but there was no significant difference in 3-UPA after treatment, suggesting that THSWD could downregulate the level of 3-UPA. Moreover, it was found for the first time that 3-UPA was positively associated with TC, suggesting that 3-UPA might be also associated with the lipid-lowering effect of THSWD.

### Limitation

Our study has some limitations. First, a small number of participants were selected in our study, which might be the reason for the statistically significant difference of metabolites between the two groups before the treatment. Second, the metabolomics itself has some limitations. A single instrument to detect all metabolites in a single analysis is not available. In the study of CHD-related metabolomics, the number of metabolites identified in serum was usually less than in cells or tissues, and the detected serum metabolites might originate from non-myocardial tissues. Moreover, the use of metabolic drugs and the excretion of liver and kidney affect the serum level of metabolites, increasing the uncertainty of applying metabolomics to the clinical detection of CHD.

## Conclusion

In conclusion, THSWD promoted energy generation by upregulating FA metabolism and downregulating glucose metabolism. THSWD also downregulated glycerophospholipid metabolism and arachidonic acid metabolism, and was involved in amino acid metabolism. This was the first study to confirm the changes of serum metabolomics in CHD patients after the treatment of THSWD. It is revealed that small molecular metabolites such as glycerophosphocholine, 8,9-DiHETrE, 5′-MTA, hippurate, indoxyl sulfate, and 3-UPA may be the potential targets of THSWD for anticoagulation and lipid reduction, and the material foundation of the “blood” to promote blood circulation in CHD treatment.

## Data Availability Statement

The datasets generated for this study are available on request to the corresponding author.

## Ethics Statement

The studies involving human participants were reviewed and approved by the Ethics Review Committee of the Chinese PLA General Hospital (No. S2015-048-01). The patients/participants provided their written informed consent to participate in this study.

## Author Contributions

XL conceived, designed, and supervised the clinical trial and experiments, and contributed to manuscript revision. TT performed the trial and statistical analysis, and drafted the manuscript. TH and HM co-performed the trial. XW co-supervised the clinical trial. All authors read and approved the submitted version.

## Funding

This work was supported by the National Natural Science Foundation of China (No. 31971049) and the National Basic Research Program of China (No. 2015CB554402, 2015CB554405).

## Conflict of Interest

The authors declare that the research was conducted in the absence of any commercial or financial relationships that could be construed as a potential conflict of interest.

## Supplementary Material

Supplementary Figure The representative MS/MS spectra of six significant metabolites (succinylcarnitine, glycerophosphocholine, succinate, hippurate, indoxyl sulfate, gluconic acid). A: The MS/MS spectra of the serum sample; B. The MS/MS spectra in the in-house library.

Supplementary Table The detail data of 513 identified metabolites.
