# Supplementary figures and images for: Non-Targeted Metabolomic Profiling of Coronary Heart Disease Patients With Taohong Siwu Decoction Treatment
Source: Front Pharmacol. 2020 May 8;11:651. doi: 10.3389/fphar.2020.00651 (PMC7227603; doi:10.3389/fphar.2020.00651)

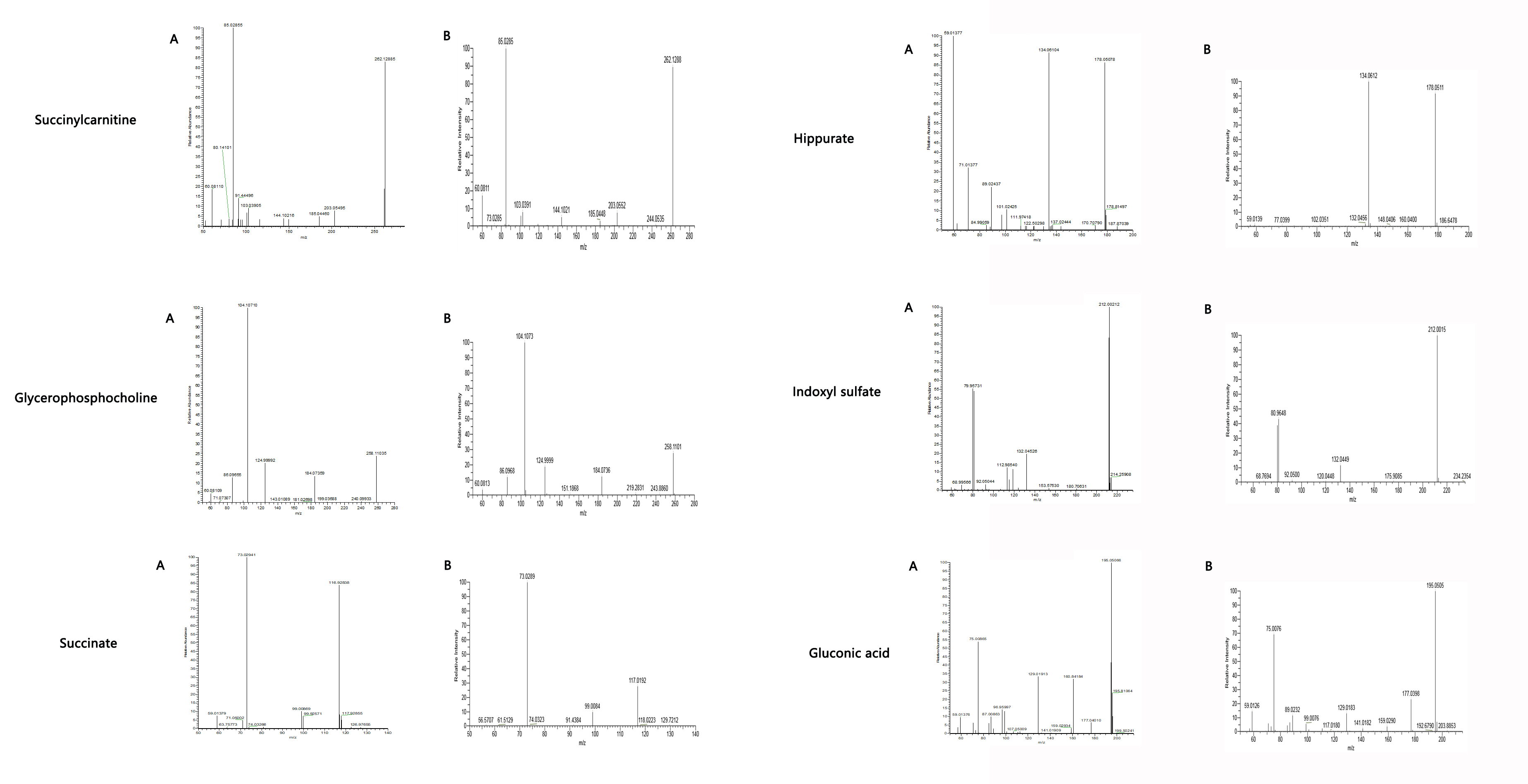

Supplement: Supplementary Figure — The representative MS/MS spectra of six significant metabolites (succinylcarnitine, glycerophosphocholine, succinate, hippurate, indoxyl sulfate, gluconic acid). A: The MS/MS spectra of the serum sample; B. The MS/MS spectra in the in-house library. [file Image_1.jpeg]
