# Supplementary material for: Non-Targeted Metabolomic Profiling of Coronary Heart Disease Patients With Taohong Siwu Decoction Treatment
Source: Front Pharmacol. 2020 May 8;11:651. doi: 10.3389/fphar.2020.00651 (PMC7227603; doi:10.3389/fphar.2020.00651)
Supplement: Supplementary Table — The detail data of 513 identified metabolites. [file DataSheet_1.pdf]

Supplementary Material

Supplementary Figure

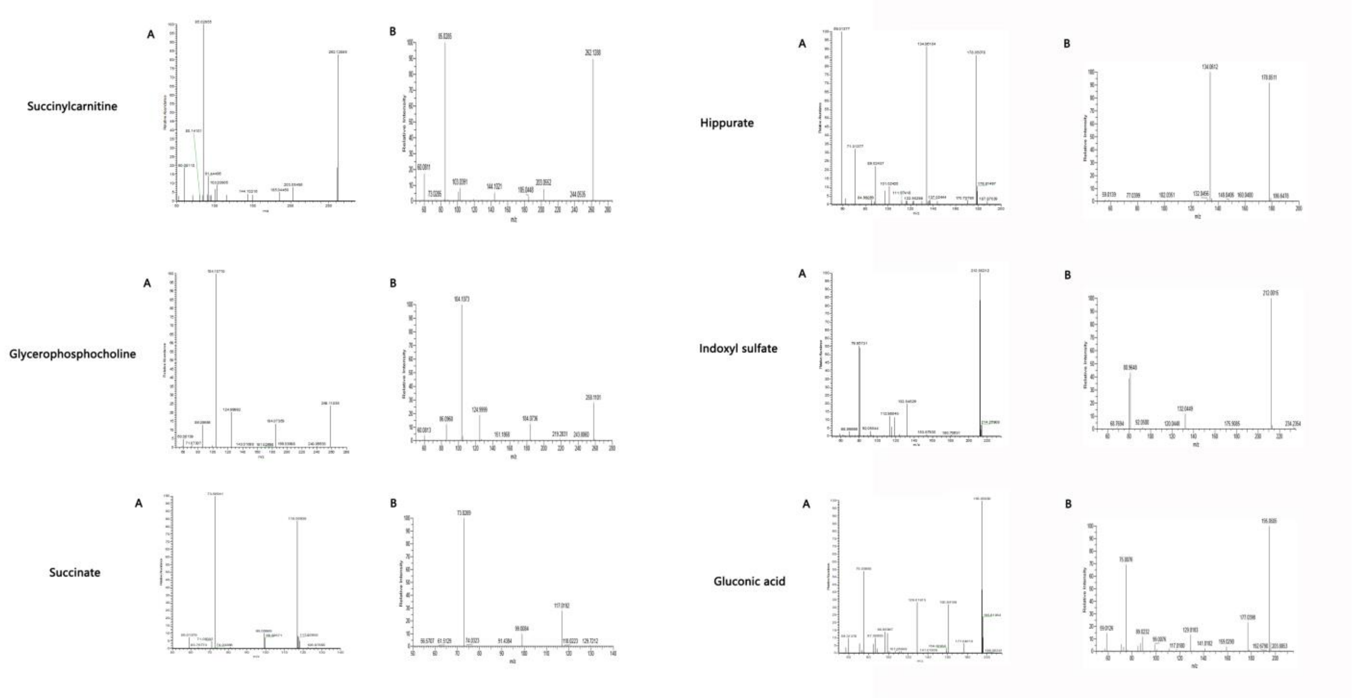

Supplementary Figure. The representative MS/MS spectra of six significant metabolites (succinylcarnitine, glycerophosphocholine, succinate, hippurate, indoxyl sulfate, Gluconic acid). A: The MS/MS spectra of the serum sample; B. The MS/MS spectra in the in-house library.

Supplementary Table

Supplementary Table. The detail data of 513 identified metabolites

No	Compound Name	ESI mode	Mass (m/z)	RT(min)
1	(3S)-3,6-Diaminohexanoate/(3S,5S)-3,5-Diaminohexanoate	neg	145.09825	1.02
2	16-Hydroxypalmitic acid	neg	271.22787	10.31
3	1H-Indole-4-carboxaldehyde	neg	144.04548	7.80

4	2,3-dihydroxybenzoate	neg	153.01881	6.47
5	2,6-Dihydroxybenzoic acid	neg	153.01933	6.47
6	2-amino-2-methylpropanoate	neg	102.05553	0.90
7	2-Dehydro-L-gulonate/3-Dehydro-L-gulonate	neg	193.03538	0.90
8	2-deoxy-D-glucose	neg	163.06068	1.04
9	2-Furoic acid	neg	111.00876	0.87
10	2-hydroxybutyric acid	neg	103.03954	18.91
11	2-Hydroxyglutarate	neg	147.0299	0.89
12	2-keto valeric acid	neg	115.04007	1.81
13	2-Phenylpropionate	neg	149.0608	6.52
14	3,4-dihydroxybenzoate	neg	153.01881	6.47
15	3,5-dimethyl-p-anisic acid	neg	179.07136	9.26
16	3-aminoisobutanoate	neg	102.05553	0.90
17	3-Furoic acid	neg	111.00876	0.87
18	3-hydroxy-3-methylglutarate	neg	161.04503	1.02
19	3-Hydroxybutyric acid	neg	103.03955	18.90
20	3-Hydroxycapric acid	neg	187.13397	7.91
21	3-Hydroxyisovaleric acid	neg	117.05517	1.46
22	3-Indoleacetic Acid	neg	174.05605	5.80
23	3-Indolepropionic acid	neg	188.0717	6.74
24	3-methyl-2-oxobutyrate	neg	115.03952	1.81

25	3-Methyl-2-oxovaleric acid/2-Ketohexanoic acid	neg	129.05572	4.29
26	3-Methylphenylacetic acid	neg	149.0608	6.90
27	3-phenylpropionate (Hydrocinnamic acid)	neg	149.06025	6.52
28	3-ureidopropionate	neg	131.04569	0.93
29	4-Coumaryl alcohol	neg	149.0608	6.52
30	4-Hydroxybenzenesulfonic acid	neg	172.9914	5.42
31	5,6-Epoxy-8,11,14-eicosatrienoic acid/5-HETE	neg	319.22787	13.12
32	5-oxo-D-proline	neg	128.03479	1.00
33	7alpha,25-Dihydroxy-4-cholesten-3-one/Calcitriol	neg	415.32177	11.67
34	8,9-DiHETrE	neg	337.23843	12.89
35	Acetoacetic acid/2-Ketobutyric acid	neg	101.02442	1.01
36	Acetyl-N-formyl-5-methoxykynurenamine	neg	263.10373	6.04
37	Adonitol	neg	151.0612	1.04
38	allothreonine	neg	118.05044	0.99
39	alpha-hydroxyisobutyric acid	neg	103.03954	18.91
40	alpha-Ketoglutaric acid (Oxoglutaric acid)	neg	145.01425	0.87
41	Alpha-Linolenic acid (18:3)	neg	277.2173	11.74
42	Aminoadipic acid	neg	160.06153	0.89
43	Arabinonic acid	neg	165.03991	0.90
44	Arabinose/Xylulose/Ribulose/Arabinose/Ribose/Xylose/Lyxose	neg	149.04555	0.89
45	Arabitol/Ribitol/Xylitol	neg	151.0612	1.04

46	Arachidic Acid (20:0)	neg	311.29555	15.77
47	Arachidonic Acid (20:4)	neg	303.23295	12.16
48	Ascorbic acid	neg	175.02429	0.92
49	Asp-Phe	neg	279.09754	2.09
50	Behenic acid (22:0)	neg	339.32631	18.10
51	beta-alanine	neg	88.03988	0.98
52	Bilirubin	neg	583.25621	10.55
53	Biliverdin	neg	581.24056	8.29
54	C-6 Ceramide	neg	396.34831	14.76
55	cholesterol sulfate	neg	465.3044	12.03
56	Cholic acid/a-TCA/b-TCA/w-TCA	neg	407.2803	8.41
57	Cinnamoylglycine	neg	204.06662	5.92
58	cis-Aconitic acid	neg	173.00864	0.87
59	Citraconic acid	neg	129.01933	0.87
60	citrate	neg	191.01921	0.89
61	creatinine	neg	112.05111	1.12
62	D-(+)-Glucose	neg	179.05611	1.00
63	D-alanine	neg	88.03988	0.98
64	D-Alpha-aminobutyric acid	neg	102.05553	0.90
65	D-Arabinono-1,4-lactone	neg	147.0299	0.88
66	D-Arabinose 5-phosphate/D-Ribulose 5-phosphate	neg	229.01188	0.89

67	D-Arabitol	neg	151.0612	1.04
68	D-aspartate	neg	132.02971	0.89
69	Deoxyadenosine monophosphate	neg	330.06037	11.90
70	Deoxyribose 1-phosphate/1-Deoxy-D-xylulose 5-phosphate	neg	213.01696	7.82
71	D-Fucose	neg	163.06119	1.04
72	D-Glucaric acid	neg	209.03029	0.87
73	D-glucono-1,5-lactone	neg	177.03994	0.89
74	D-Glucuronic acid	neg	193.03538	0.90
75	D-glucuronolactone	neg	175.02429	0.92
76	D-Glutamic acid	neg	146.04536	0.89
77	D-Glutamylglycine	neg	203.06735	0.89
78	dihydroxymandelic acid	neg	183.02938	1.65
79	DL-2-Aminooctanoic acid	neg	158.11865	6.66
80	Docosahexaenoic acid (22:6)	neg	327.23295	11.87
81	D-saccharic acid	neg	209.02977	0.87
82	D-tryptophan	neg	203.08208	5.86
83	D-Xylitol	neg	151.0612	1.04
84	D-Xylulose 5-phosphate	neg	229.01188	0.89
85	Eicosanoic acid (20:1)	neg	309.27936	14.21
86	Eicosapentaenoic acid (20:5)	neg	301.2173	11.59
87	FA(17:1)-H	neg	267.2328	12.60

88	FA(20:2)-H	neg	307.2643	13.25
89	FA(20:3)-H	neg	305.2483	12.60
90	FA(22:1)-H	neg	337.3117	15.75
91	FA(22:2)-H	neg	335.2962	14.33
92	FA(22:3)-H	neg	333.2804	13.59
93	FA(22:4)-H	neg	331.2642	12.83
94	FA(22:5)-H	neg	329.2484	12.35
95	FA(23:1)-H	neg	351.3272	16.26
96	FA(24:1)-H	neg	365.3425	17.92
97	FA(24:2)-H	neg	363.3276	15.83
98	FA(24:4)-H	neg	359.2962	13.68
99	FA(24:6)-H	neg	355.2646	12.58
100	FA(26:4)-H	neg	387.327	14.73
101	fumarate	neg	115.00316	0.88
102	galactarate	neg	209.02977	0.87
103	Galactitol	neg	181.07124	1.00
104	galacturonic acid	neg	193.03485	0.90
105	gama-Aminobutryic acid (GABA)	neg	102.05605	0.90
106	Gamma Glutamylglutamic acid	neg	275.08847	0.88
107	Gln-Glu	neg	274.10281	0.89
108	Gluconic acid	neg	195.0505	0.90

109	Gluconolactone	neg	177.03994	0.89
110	Glu-Gln	neg	274.10086	0.89
111	Glu-Gly	neg	203.0657	0.89
112	Glu-Leu	neg	259.1283	2.22
113	Glutarate semialdehyde	neg	115.04007	1.81
114	Glutaric acid	neg	131.03446	1.01
115	Glyceric acid	neg	105.01933	0.90
116	glycerol 2-phosphate	neg	171.00587	0.89
117	Glycerol 3-phosphate	neg	171.0064	0.88
118	Glycochenodeoxycholic acid/GUDCA	neg	448.30685	8.48
119	Glycocholic Acid	neg	464.30176	8.08
120	Gly-Glu	neg	203.0657	0.89
121	guanidinoacetate	neg	116.04603	1.68
122	Heptadecanoic acid (17:0)	neg	269.24806	13.55
123	Hexadecanedioic acid	neg	285.20713	8.47
124	Hexylresorcinol	neg	193.1234	8.76
125	hippurate	neg	178.05097	4.85
126	Histidine	neg	154.06168	1.07
127	Hydrocinnamic acid	neg	149.0608	6.52
128	Hydroxycaprylic acid	neg	159.10267	6.83
129	Hydroxyphenyllactic acid	neg	181.05008	1.51

130	hydroxypyruvate	neg	103.00316	19.02
131	Iditol/Mannitol/Dulcitol	neg	181.07176	1.61
132	Ile-Glu	neg	259.12939	2.22
133	Ile-Ile	neg	243.17032	6.67
134	Ile-Leu	neg	243.17032	6.67
135	Indole	neg	116.05057	5.84
136	Indoleacetic acid	neg	174.0555	5.76
137	Indoleacrylic acid	neg	186.05605	6.50
138	Indoxyl sulfate	neg	212.00178	6.18
139	isocitric acid	neg	191.01921	0.89
140	Itaconic acid	neg	129.01933	0.87
141	Ketoleucine	neg	129.05517	4.29
142	L-(+)-2-Aminobutyric acid	neg	102.05605	0.90
143	lactate	neg	89.0239	0.95
144	L-alanine	neg	88.03988	0.98
145	L-Arabitol	neg	151.0612	1.04
146	L-Arginine	neg	173.1044	1.05
147	L-Asparagine	neg	131.04569	0.93
148	L-aspartate	neg	132.02971	0.90
149	L-Aspartyl-L-phenylalanine	neg	279.09864	2.09
150	Lauric acid(12:0)	neg	199.1698	10.68

151	Leucinic acid; Hydroxyisocaproic acid	neg	131.07137	2.78
152	Leu-Glu	neg	259.12939	9.40
153	Leu-Ile	neg	243.17032	6.67
154	Leukotriene A4	neg	317.21222	13.11
155	Leu-Leu	neg	243.17032	6.67
156	Leu-Phe	neg	277.15467	7.08
157	L-glutamic acid	neg	146.04536	0.90
158	L-Glutamine	neg	145.06134	0.93
159	Linolenic acid (18:2)	neg	279.23295	12.34
160	L-Isoleucine/L-Leucine	neg	130.08683	1.72
161	L-kynurenine	neg	207.07699	3.46
162	L-lysine	neg	145.09773	1.02
163	L-methionine	neg	148.04325	1.40
164	L-norvaline	neg	116.07118	1.13
165	L-Phenylalanine	neg	164.07118	3.54
166	L-proline	neg	114.05553	1.06
167	L-rhamnose	neg	163.06067	1.04
168	L-serine	neg	104.03479	0.93
169	L-threonine	neg	118.05044	0.99
170	L-tryptophan	neg	203.08208	5.85
171	L-Tyrosine	neg	180.06609	1.48

172	L-Xylonate	neg	165.03991	0.90
173	Maleic acid	neg	115.00316	0.88
174	Malic acid	neg	133.01373	0.88
175	malonate	neg	103.00316	19.02
176	Methylmalonic acid	neg	117.01881	0.88
177	Myristic acid (14:0)	neg	227.20111	11.91
178	N-Acetylaspartylglutamic acid	neg	303.08339	1.73
179	N-Acetylglutamic acid	neg	188.05645	0.88
180	N-acetyl-L-aspartic acid	neg	174.04027	0.88
181	N-Acetylneuraminic acid	neg	308.09818	0.89
182	N-Acetylserine	neg	146.04536	0.90
183	N-alpha-acetyl-L-asparagine	neg	173.05626	18.92
184	Nicotinic acid	neg	122.02423	19.05
185	N-methyl-L-glutamate	neg	160.06101	0.89
186	N-methyl-L-histidine	neg	168.07785	1.52
187	Oenanthic ether	neg	157.1234	8.79
188	Oleic Acid/Elaidic Acid/Vaccenic acid/Petroselinic acid (18:1)	neg	281.2486	13.11
189	Palmitic acid (16:0)	neg	255.23295	12.95
190	Palmitoleic acid/Gaidic acid (16:1)	neg	253.2173	12.14
191	Pantothenic Acid	neg	218.1034	1.83
192	p-Cresol sulfate	neg	187.0065	6.91

193	Pelargonic acid (9:0)	neg	157.1234	8.78
194	Pentacosylic acid (25:0)	neg	381.37326	15.56
195	Pentadecanoic acid (15:0)	neg	241.2173	12.47
196	Phe-Asp	neg	279.09754	2.25
197	Phe-Ile	neg	277.15467	7.09
198	Phe-Leu	neg	277.15412	7.09
199	phenol sulfate	neg	172.99085	5.42
200	Phe-Phe	neg	311.13902	7.36
201	Phe-Trp	neg	350.14991	7.44
202	Phosphoric acid	neg	96.96962	0.94
203	Phytanic Acid	neg	311.29555	15.77
204	Picolinic acid	neg	122.02475	19.05
205	Pipecolic acid	neg	128.07118	1.40
206	prasterone sulfate	neg	367.15792	8.32
207	Pregnan-20-one, 17-(acetyloxy)-3-hydroxy-6-methyl-, (3b,5b,6a)-/Pregnan-20-one, 17-(acetyloxy)-3-hydroxy-6-methyl-, (3a,5b,6a)-	neg	389.26973	10.47
208	Pregnenolone	neg	315.23295	11.96
209	Prostaglandin G2	neg	367.21261	10.94
210	Pyroglutamic acid	neg	128.03532	1.00
211	Pyrrolidonecarboxylic acid	neg	128.03532	1.00
212	Pyruvic acid	neg	87.00877	1.00
213	ribose 5-phosphate	neg	229.01136	0.89

214	Sebacic acid	neg	201.11323	5.84
215	Stearic acid (18:0)	neg	283.26425	14.19
216	Stearic Acid ethyl ester	neg	311.29555	15.77
217	Stearidonic acid (18:4)	neg	275.20111	11.19
218	succinate	neg	117.01881	0.88
219	succinate semialdehyde	neg	101.0239	1.01
220	Sulfate	neg	96.9601	0.87
221	Sulfoacetate	neg	138.97067	0.88
222	Tagatose/Gulose/Fructose/Galactose/Mannose/Sorbose/Allose	neg	179.05611	1.00
223	Taurine	neg	124.00687	0.96
224	Taurochenodeoxycholic acid	neg	498.28948	8.59
225	Tetracosanoic acid (24:0)	neg	367.35815	14.85
226	Tetradecanedioic acid	neg	257.17583	7.60
227	Threonic acid	neg	135.0299	0.90
228	trans-aconitate	neg	173.00864	0.87
229	Tricosylic acid (23:0)	neg	353.34196	18.52
230	Trimethylacetic acid	neg	101.06025	2.21
231	Trp-Phe	neg	350.14991	7.45
232	Undecanoic acid (11:0)	neg	185.1547	10.04
233	Uric acid	neg	167.02054	1.00
234	Uridine	neg	243.06174	1.90

235	Xanthine	neg	151.02563	1.62
236	(25R)-5beta-cholestane-3alpha,7alpha,12alpha,26-tetrol	pos	437.36254	0.84
237	13Z-Docosenamide/13E-Docosenamide	pos	338.34174	0.85
238	1-aminocyclopropane-1-carboxylate	pos	102.05548	9.73
239	1-methyladenosine	pos	282.12021	8.73
240	1-Methylhistamine	pos	126.10257	8.81
241	1-Methylnicotinamide	pos	137.07094	6.66
242	2,6 dimethylheptanoyl carnitine	pos	302.23261	3.86
243	2-amino-2-methylpropanoate	pos	104.07113	9.12
244	2-Aminobenzoic acid/p-Aminobenzoic acid	pos	138.05495	8.68
245	2-Aminobutyric acid	pos	104.07061	9.12
246	2-Ethylacrylylcarnitine, Tiglylcarnitine	pos	244.15436	6.02
247	2-Hydroxyadenine	pos	152.05669	7.03
248	2-Methylbutyroylcarnitine	pos	246.17001	5.52
249	2-Methylbutyroylcarnitine; 3-Methylbutyrylcarnitine	pos	246.16998	5.52
250	2-Methylbutyrylglycine	pos	160.09737	9.06
251	2'-O-Methyladenosine	pos	282.11968	8.75
252	2-Pyrrolidinone	pos	86.06004	1.40
253	3 alpha,7 alpha,26-Trihydroxy-5beta-cholestane	pos	421.36762	0.84
254	3a,7a,12a-Trihydroxy-5b-cholestanoic acid	pos	451.3418	0.90
255	3a,7a-Dihydroxy-5b-cholestane	pos	405.37271	0.88

256	3alpha,7alpha-Dihydroxy-5beta-cholestanate	pos	435.34689	0.88
257	3-amino-4-hydroxybenzoic acid	pos	154.05039	2.03
258	3-aminoisobutanoate	pos	104.07113	9.12
259	3-Hydroxy-2-methylpyridine-5-carboxylate	pos	154.04987	2.03
260	3-Hydroxyanthranilic acid	pos	154.04987	2.03
261	3-Hydroxyisovalerylcarnitine	pos	262.16492	8.52
262	3-hydroxy-N6,N6,N6-trimethyl-L-lysine	pos	205.15467	11.29
263	3-ketosphingosine	pos	298.27406	0.87
264	3-methyglutaric acid	pos	147.06571	11.43
265	3-methylhistamine	pos	126.1031	8.81
266	3-Methylhistidine	pos	170.0924	11.15
267	4,8 dimethylnonanoyl carnitine	pos	330.26391	3.14
268	4-Guanidinobutanoic acid	pos	146.0924	8.73
269	4-hydroxy-2-quinolinecarboxylic acid	pos	190.05039	6.80
270	4-Pyridoxic Acid	pos	184.06096	2.41
271	4-Pyrimidine Methanamine / 2-Aminomethylpyrimidine	pos	110.07127	11.13
272	5-Acetamidovalerate	pos	160.09682	9.06
273	5-Hydroxyindoleacetic acid	pos	192.06552	7.97
274	5-L-Glutamyl-L-alanine	pos	219.09755	10.53
275	5-methylcytosine	pos	126.06671	6.34
276	5'-Methylthioadenosine	pos	298.09736	11.32

277	5-oxo-proline	pos	130.05039	10.06
278	7a,12a-Dihydroxy-5b-cholestan-3-one	pos	419.35197	0.86
279	7alpha,25-Dihydroxy-4-cholesten-3-one/Calcitriol	pos	417.33632	0.86
280	7-Methylguanine	pos	166.07234	5.22
281	acetoacetate	pos	103.03897	9.15
282	Acetylcholine	pos	146.11756	9.08
283	Acetyl-N-formyl-5-methoxykynurenamine	pos	265.11828	6.63
284	Ala-Glu	pos	219.0981	10.53
285	Ala-His	pos	227.11496	11.33
286	allothreonine	pos	120.06604	9.75
287	alpha-glutamylalanine	pos	219.0981	10.53
288	Alpha-Linolenic acid	pos	279.23186	0.89
289	Alpha-Tocopherol	pos	431.38836	0.81
290	Anthranilate	pos	138.05548	8.68
291	Arg-Asn	pos	289.16297	12.64
292	Arg-Glu	pos	304.16318	12.49
293	Arg-Pro	pos	272.17281	11.54
294	Arg-Val	pos	274.18846	11.14
295	Asn-Arg	pos	289.16297	12.64
296	Asn-Val	pos	232.13028	9.76
297	Asp-Phe	pos	281.11429	9.74

298	Asp-Thr	pos	235.09356	11.32
299	Asymmetric dimethylarginine	pos	203.15025	10.71
300	beta-Alanine	pos	90.05548	9.56
301	beta-D-Glucosamine	pos	180.08718	9.93
302	Betaine	pos	118.08678	8.28
303	Betaine aldehyde	pos	102.09134	9.15
304	Bilirubin	pos	585.27076	0.85
305	Biliverdin	pos	583.25511	1.89
306	Butyrylcarnitine	pos	232.15433	6.31
307	caffeine	pos	195.08818	1.01
308	Carnosine	pos	227.11439	11.13
309	Chenodeoxycholic acid glycine conjugate	pos	450.3214	4.76
310	Cholesterol-H2O	pos	369.35155	0.83
311	Choline	pos	104.10699	4.96
312	cis-5-Tetradecenoylcarnitine	pos	370.29521	3.31
313	Citrulline	pos	176.10349	10.29
314	Creatine	pos	132.07728	9.55
315	Creatinine	pos	114.06671	4.51
316	Cytosine	pos	112.05106	5.02
317	D-(+)-Glucose	pos	181.07067	1.53
318	D-Alpha-aminobutyric acid	pos	104.07061	9.12

319	Decanoylcarnitine	pos	316.24824	3.77
320	Delta-Tocopherol/Cholest-5-ene-3beta,26-diol	pos	403.35706	0.88
321	deoxycarnitine	pos	146.11808	9.08
322	D-Galactose	pos	181.07067	1.53
323	D-Glutamylglycine	pos	205.0819	10.95
324	Diaminopimelic acid	pos	191.10264	10.04
325	diethanolamine	pos	106.08678	8.05
326	Dimethylglycine	pos	104.07061	9.12
327	Dimethylurea	pos	89.07094	1.48
328	DL-2-Aminooctanoic acid	pos	160.13321	7.25
329	DL-Stearoylcarnitine	pos	428.37346	2.75
330	D-Mannosamine	pos	180.08718	9.93
331	Dodecanoylcarnitine	pos	344.27954	3.25
332	Elaidic carnitine,Vaccenyl carnitine	pos	426.35781	2.98
333	Epinephrine	pos	184.09734	9.56
334	Ergothioneine	pos	230.09577	9.30
335	Estrone glucuronide	pos	447.20134	8.12
336	Flavone	pos	223.07536	0.83
337	galactosamine	pos	180.08718	9.93
338	Gama-Aminobutryic acid	pos	104.07061	9.21
339	Gln-Glu	pos	276.11956	11.30

340	Glu-Ala	pos	219.0981	10.51
341	Glu-Arg	pos	304.16209	12.55
342	Glucosamine	pos	180.08718	9.93
343	Glu-Gln	pos	276.11761	11.32
344	Glu-Gly	pos	205.08245	10.72
345	Glu-Ile	pos	261.14505	9.61
346	Glu-Leu	pos	261.14505	9.61
347	Glu-Lys	pos	276.15595	12.58
348	Glu-Met	pos	279.10147	9.99
349	Glu-Phe	pos	295.1294	9.69
350	Glu-Ser	pos	235.09301	11.32
351	Glutamylphenylalanine	pos	295.1294	9.69
352	Glu-Val	pos	247.1294	9.96
353	Glycerophosphocholine	pos	258.1101	10.39
354	Glycine	pos	76.03983	9.85
355	Glycocholic Acid	pos	466.31631	7.13
356	Gly-Glu	pos	205.08245	10.95
357	Gly-Thr	pos	177.08808	10.34
358	Gly-Val	pos	175.10881	9.49
359	Guanidineacetic acid	pos	118.06163	9.65
360	Guanine	pos	152.05721	7.03

361	Hexanoylcarnitine	pos	260.18563	4.99
362	Hexanoylglycine	pos	174.11302	8.40
363	Hexylresorcinol	pos	195.13796	0.87
364	Hippuric acid	pos	180.06552	2.85
365	His-Ala	pos	227.11496	11.29
366	His-Ile	pos	269.16191	9.84
367	His-Leu	pos	269.16191	9.84
368	His-Phe	pos	303.14626	9.75
369	Histamine	pos	112.08692	9.92
370	His-Trp	pos	342.15716	9.73
371	homoserine	pos	120.06604	9.75
372	Hydroxybutyrylcarnitine	pos	248.1498	9.07
373	Hydroxycapric acid-H2O	pos	171.1385	0.88
374	Hypotaurine	pos	110.02755	9.53
375	Hypoxanthine	pos	137.04631	4.46
376	Ile-Ala	pos	203.14011	9.18
377	Ile-Glu	pos	261.14614	9.60
378	Ile-Ile	pos	245.18706	6.89
379	Ile-Leu	pos	245.18706	6.89
380	Ile-Phe	pos	279.17141	6.32
381	Ile-Val	pos	231.17141	7.92

382	Imidazoleacetic acid	pos	127.0502	9.42
383	Iminodiacetic acid	pos	134.04479	10.78
384	Indoleacetaldehyde	pos	160.07569	6.13
385	Indoleacrylic acid	pos	188.07061	7.90
386	Inosine	pos	269.08857	6.78
387	Isobutyryl carnitine	pos	232.1544	6.31
388	Ketoleucine	pos	131.07082	9.73
389	L-Acetylcarnitine	pos	204.12303	8.38
390	L-Alanine	pos	90.05495	9.57
391	L-Alpha-aminobutyric acid (homoalanine)	pos	104.07061	9.14
392	L-Arginine	pos	175.11948	11.42
393	L-Asparagine	pos	133.06132	10.17
394	L-Aspartic Acid	pos	134.04531	10.78
395	L-Aspartyl-L-phenylalanine	pos	281.1132	9.74
396	Lathosterol-H2O	pos	369.35155	0.83
397	lauroylcarnitine	pos	344.28005	3.25
398	L-Carnitine	pos	162.11247	9.15
399	L-Cystine	pos	241.03165	12.53
400	Leu-Glu	pos	261.14614	9.61
401	Leu-His	pos	269.16191	9.84
402	Leu-Ile	pos	245.18706	6.89

403	Leu-Leu	pos	245.18706	6.89
404	Leu-Phe	pos	279.17141	6.29
405	Leu-Val	pos	231.17141	7.90
406	L-gamma-glutamyl-L-isoleucine	pos	261.14505	9.61
407	L-gamma-glutamyl-L-leucine	pos	261.14505	9.61
408	L-gamma-glutamyl-L-valine	pos	247.1294	9.96
409	L-Glutamic acid	pos	148.06096	10.24
410	L-Glutamine	pos	147.07694	10.06
411	L-Histidine	pos	156.07727	11.32
412	L-Homocitrulline/N6-Carbamoyl-DL-Lysine	pos	190.11917	10.10
413	Linoleic acid	pos	281.24751	0.88
414	Linoleoyl Ethanolamide	pos	324.28971	0.88
415	Linoleyl carnitine, Linoelaidyl carnitine; acylcarnitine C18:2	pos	424.34216	3.12
416	L-Isoleucine	pos	132.10243	8.45
417	L-Kynurenine	pos	209.09259	7.96
418	ll-2,6-diaminoheptanedioate	pos	191.10316	10.04
419	L-Leucine	pos	132.10243	8.07
420	L-Lysine	pos	147.11333	11.63
421	L-Methionine	pos	150.05885	8.76
422	L-Methionine S-oxide	pos	166.05324	10.03
423	L-Octanoylcarnitine	pos	288.21696	4.42

424	L-Palmitoylcarnitine	pos	400.34214	2.93
425	L-Phenylalanine	pos	166.08678	7.92
426	L-Pipecolic acid-1	pos	130.08678	8.13
427	L-pipecolic acid-2	pos	130.08678	11.43
428	L-Proline	pos	116.07113	9.21
429	L-Serine	pos	106.04987	10.11
430	L-threonine	pos	120.06604	9.75
431	L-Threonine/L-Homoserine	pos	120.06552	9.75
432	L-tryptophan	pos	205.09768	7.92
433	L-Tyrosine	pos	182.08169	9.15
434	L-Valine	pos	118.08678	9.22
435	Lys-Glu	pos	276.15704	12.54
436	LysoPC(20:0)	pos	552.40237	5.90
437	LysoPC(20:1)	pos	550.38672	5.94
438	Malonylcarnitine	pos	248.11288	10.25
439	Met-Glu	pos	279.10256	9.99
440	METHACHOLINE-like	pos	160.13321	7.25
441	Methylimidazoleacetic acid	pos	141.06585	9.34
442	methyl-L-histidine	pos	170.09293	11.15
443	mevalolactone	pos	131.0708	9.73
444	Myristoylcarnitine	pos	372.31084	3.39

445	N??-Acetyl-L-lysine	pos	189.12337	9.83
446	N1-Acetylspermidine	pos	188.17574	10.55
447	N6,N6,N6-Trimethyl-L-lysine	pos	189.15975	11.25
448	N-acetyl-DL-serine	pos	148.06096	10.24
449	N-Acetylglycine	pos	118.04987	8.29
450	N-acetylhistidine	pos	198.08787	10.31
451	N-acetylleucine	pos	174.11302	8.40
452	N-Acetylornithine	pos	175.10772	9.75
453	N-Acetylputrescine	pos	131.11789	7.96
454	N-Alpha-acetyllysine	pos	189.12337	9.61
455	Niacinamide	pos	123.05584	1.55
456	Niacinamide (VB3)	pos	123.05581	1.55
457	Nicotine	pos	163.12297	5.20
458	N-Palmitoylsphingosine	pos	538.51938	0.84
459	O-Acetyl-L-carnitine	pos	204.12303	8.38
460	O-Acetyl-L-serine	pos	148.06096	10.24
461	Oleamide	pos	282.27914	0.84
462	Oleoyl Ethanolamide	pos	326.30535	0.87
463	Ornithine	pos	133.09767	11.80
464	Palmitic amide	pos	256.26349	0.86
465	palmitoyl sphingomyelin (SM(d18:1/16:0))	pos	703.5754	5.70

466	Pantothenic Acid	pos	220.11795	3.18
467	paraxanthine	pos	181.07253	1.53
468	Phe-Asp	pos	281.11429	9.74
469	Phe-Glu	pos	295.1294	9.69
470	Phe-His	pos	303.14626	9.75
471	Phe-Ile	pos	279.17141	6.35
472	Phe-Leu	pos	279.17087	6.35
473	Phe-Phe	pos	313.15576	6.43
474	Phe-Trp	pos	352.16666	6.54
475	Phosphorylcholine	pos	184.07332	5.83
476	Phytosphingosine	pos	318.30027	2.33
477	Pivaloylcarnitine	pos	246.17001	5.52
478	Progesterone	pos	315.23186	3.94
479	Propionylcarnitine	pos	218.13868	7.16
480	Pro-Val	pos	215.14011	9.25
481	Putrescine	pos	89.10785	11.30
482	Pyroglutamic acid (5-oxoproline)	pos	130.04987	10.06
483	Pyrrole-2-carboxylic acid	pos	112.03931	6.25
484	Pyrrolidonecarboxylic acid	pos	130.04987	10.06
485	Ribothymidine	pos	259.09246	9.73
486	Ser-Ala	pos	177.08808	10.34

487	Ser-Glu	pos	235.0941	11.32
488	Serotonin	pos	177.10224	6.12
489	Shikimic Acid	pos	175.0601	11.29
490	Spermidine	pos	146.16517	7.49
491	Spermine	pos	203.22302	8.76
492	Sphinganine	pos	302.30536	2.56
493	Succinylcarnitine	pos	262.12906	9.46
494	Taurine	pos	126.02247	9.19
495	Tetradecanoylcarnitine	pos	372.31086	3.39
496	theobromine	pos	181.07253	1.53
497	Theophylline	pos	181.07201	1.53
498	Thr-Gly	pos	177.08808	10.34
499	Thymine	pos	127.05073	9.42
500	trans-Hexadec-2-enoyl carnitine	pos	398.32651	3.16
501	Trigonelline	pos	138.05548	8.68
502	Trimethylamine N-oxide (TMAO)	pos	76.07624	5.39
503	Trp-His	pos	342.15716	9.73
504	Trp-Phe	pos	352.16666	6.54
505	Tyr-Arg	pos	338.18337	11.27
506	Uric acid	pos	169.03562	1.51
507	Urocanic acid	pos	139.05073	3.27

508	Val-Arg	pos	274.18846	10.96
509	Val-Asn	pos	232.13028	9.78
510	Val-Glu	pos	247.13049	9.98
511	Val-Ile	pos	231.17141	7.92
512	Val-Leu	pos	231.17141	7.93
513	Val-Pro	pos	215.14011	9.25

ESI: electrospray ionization; RT: retention time; Pos: positive; Neg: negative
